# Targeted recombination between homologous chromosomes for precise breeding in tomato

**DOI:** 10.1038/ncomms15605

**Published:** 2017-05-26

**Authors:** Shdema Filler Hayut, Cathy Melamed Bessudo, Avraham A. Levy

**Affiliations:** 1 Department of Plant and Environmental Sciences, Weizmann Institute of Science, Rehovot 76100, Israel

## Abstract

Homologous recombination (HR) between parental chromosomes occurs stochastically. Here, we report on targeted recombination between homologous chromosomes upon somatic induction of DNA double-strand breaks (DSBs) via CRISPR-Cas9. We demonstrate this via a visual and molecular assay whereby DSB induction between two alleles carrying different mutations in the *PHYTOENE SYNTHASE* (*PSY1*) gene results in yellow fruits with wild type red sectors forming via HR-mediated DSB repair. We also show that in heterozygote plants containing one *psy1* allele immune and one sensitive to CRISPR, repair of the broken allele using the unbroken allele sequence template is a common outcome. In another assay, we show evidence of a somatically induced DSB in a cross between a *psy1* edible tomato mutant and wild type *Solanum pimpinellifolium*, targeting only the *S. pimpinellifolium* allele. This enables characterization of germinally transmitted targeted somatic HR events, demonstrating that somatically induced DSBs can be exploited for precise breeding of crops.

## Abstract (web summary)

Targeted homologous recombination between parental chromosomes could facilitate precision breeding of crop plants. Here, Filler Hayut *et al*. show that CRISPR-Cas9 can be used to induce DNA double strand breaks in somatic tissue and achieve targeted recombination between homologs at an endogenous locus in tomato.

DNA double-strand breaks (DSBs) are one of the powerful forces that shape plant genomes. These DSBs may occur throughout the plant life cycle, in somatic or meiotic cells, spontaneously during the movement of replication forks or developmentally controlled as in the early stages of first meiosis^1^,^2^,^3^. They also may be induced through ionizing radiation, genotoxic drugs or through the activation of endonucleases^4^. Unrepaired DNA DSB may cause extreme types of damage including chromosome loss, leading to gamete sterility or cell death. Repair of DSBs may also be associated with insertion/deletion (indels) mutations. DSBs repair mechanisms are therefore essential for the maintenance of genome integrity. Understanding these mechanisms is critical for our ability to precisely engineer genomes, for example, for targeted mutagenesis, gene targeting or for other types of targeted chromosomes reshuffling^5^.

DNA DSB repair mechanisms have been widely studied in many organisms, including plants^6^,^7^,^8^,^9^,^10^. Studies in plants have characterized the genes involved in DSB repair via Non-Homologous-End-Joining (NHEJ) or Homologous Recombination (HR) and tested the outcome of DSB repair in both somatic and meiotic tissues (See refs 9, 10, 11, 12 for review). NHEJ has been characterized in a broad range of species and tissues (mostly somatic), using multiple DSB inducing agents including site specific meganucleases^13^, transposon excision^14^ and custom-designed nucleases, such as zinc-finger nucleases^15^, transcription activator-like effector nucleases (TALENs)^16^ and Clustered Regulatory Interspaced Short Palindromic Repeat associated protein Cas9 (CRISPR-Cas)^17^,^18^,^19^,^20^. The emerging picture from these works suggests that NHEJ is a prominent repair pathway in somatic cells. This error-prone mechanism involves indels ranging from a few base pairs (bp) to several Kbps^21^ at the DSB site and is often associated with microhomologies^22^,^23^. In addition, CRISPR-Cas-based systems prove to be highly efficient in a broad range of plant species^24^ including tomato^25^,^26^.

Several studies that addressed the mechanism of DSB repair via HR in somatic tissues were done in *Arabidopsis*, using transgenic assays that tested repair mechanisms such as intrachromosomal recombination, unequal crossover and so on. In all cases, DSB induction enhanced HR-repair rates^27^,^28^. Recombination rates from the unequal crossover assay were much lower than for intrachromosomal recombination^29^. Somatic DSB repair by an homologous chromosome, using an allelic sequence, was also studied in transgenic tobacco plants, using transposable element-induced breaks: HR repair occurred upon excision of the transposon; but was not detected with an immobile element^30^. DSB induction could also trigger HR-mediated repair using an ectopic genomic sequence template, albeit at very low frequencies^14^,^31^.

DSB induction of HR between endogenous (non-transgenic) recombination partners was shown in maize upon excision of the *Activator* (*Ac*)^32^, or the *Mutator* elements^33^. In both cases recombination occurred in *cis*, between repeats flanking the transposon in somatic tissues. By contrast, germinal *Ac* activity did not stimulate the rate of meiotic recombination between homologous chromosomes at the maize *bronze* locus^34^. This surprising result, which goes against a large body of studies in plants (see above) and other organisms^35^,^36^, might be due to a lack of coordination between Ac excision and meiotic recombination, a preference of meiotic HR for Spo11-induced breaks or another unknown reason. The ability to induce HR between homologous chromosomes at a specific genomic location would provide geneticists and breeders with a powerful tool for the targeted induction of crossover or gene conversion.

In this study, we tested whether targeted recombination between homologous chromosomes can be achieved in plants. For this purpose, we combined a series of new technologies, such as CRISPR-Cas, whole genome sequences, High-Throughput sequencing and a variety of mutants in tomato fruit colour, enabling both identification and quantification of resulting recombination products. We provide evidence for the occurrence of induced allele-dependent DSB repair in tomato, including the characterization of germinally transmitted HR events.

## Results

### Tomato fruit colour assay for the analysis of DNA DSB repair

To estimate the rate of somatic NHEJ versus HR based DSB repair at an endogenous plant locus, we designed a series of fruit colour assays. These assays enabled us to measure only those events of NHEJ that are error-prone as we cannot detect events of precise end-joining which might occur^21^,^37^. Similarly, we were only able to detect the HR events between polymorphic homologous chromosomes, while recombination between sister chromatids, which is also known to take place^29^,^38^ could not be detected.

**Figure 1:**
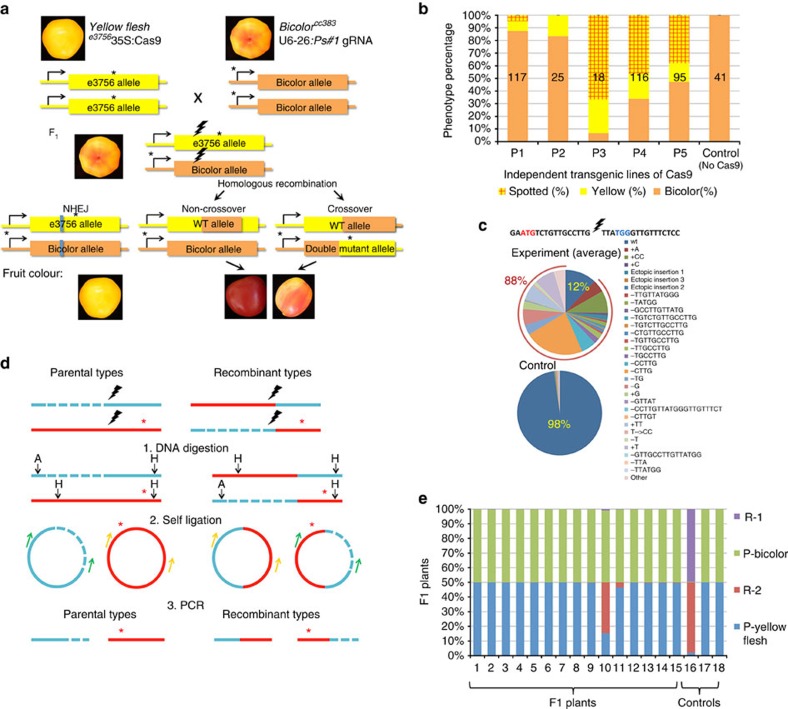
Tomato fruit colour DSB repair assay. (**a**) Crossing *yellow flesh e*^*3756*^ 35S:Cas9 and *bicolor*^*cc383*^ u6-26:Ps#1-sgRNA gives F_1_ plants with a pale Bicolour fruit phenotype. F_1_ plants expressing both Cas9 and gRNA were selected. The gRNA was designed for DSB induction (black lightning) in both alleles between the *yellow flesh e*^*3756*^ and *bicolor*^*cc383*^ mutations (*). In case of error-prone NHEJ repair (blue line) of *bicolor*^*cc383*^, fruit colour is yellow. In cases of non-crossover or crossover, fruit colour is expected to be red or bicolour or yellow with red spots in case of late event. Note that whole red fruits were not obtained. Rather, fruits with red spots in a yellow or bicolour background were found and are shown together with additional products of HR-induced repair in Supplementary Fig. 1. (**b**) Fruit phenotype distribution in F_1_ plants and control: Bicolour fruits are shown as orange boxes; Yellow fruits as yellow; Fruits with red sectors (putative somatic HR) are shown as red-stripped boxes. Each column represents a fruit population derived from cross of independent transgenic lines of Cas9 and a given u6-26:Ps#1-sgRNA line. The number of fruits analysed is shown on the column in black. (**c**) Sequences of the NHEJ DSB repair footprints and their relative frequency are shown. The CRISPR-Cas target sequence from the *PSY1* is shown on the top. The *PSY1* start codon is shown in red and the PAM in blue. The top pie represents an average of illumina Hiseq reads from 22 different F_1_ plants of the cross of *yellow flesh e*^*3756*^ 35S:Cas9 × *bicolor*^*cc383*^ u6-26:Ps#1-sgRNA. The low pie represents an average of ilummina Hiseq reads from two plants of control F_1_ population (*yellow flesh e*^*3756*^ × *bicolor*^*cc383*^ F_1_ plants with no CRISPR-Cas). (**d**) Inverse PCR scheme for identification of recombinant DNA fragments (details in Supplementary Fig. 4). (1) DNA from separate leaves was digested with ApaI(A) and HindIII(H) and then blunted. (2) Each sample was self-ligated, and (3) amplified by two different primer sets (green and yellow). Blue- *Bicolor*; red- *Yellow flesh*; Dashed blue line- *Bicolor* deletion, *- *Yellow flesh* mutation. (**e**) Ratio of parental (P) versus recombinant (R) types (obtained from panel C) in individual plants. Plants 1–15- F_1_ plants of the cross of *yellow flesh e*^*3756*^ 35S:Cas9 × *bicolor*^*cc383*^ u6-26:Ps#1-sgRNA; Plant 16- synthetic crossover control; Plants17–18-Yellow flesh × Bicolor (Cas9-) F_1_ plants.

For the first fruit colour assay, we used two mutant lines of tomato, each with a different mutation in the *Phytoene synthase 1* (*PSY1*) gene. The *yellow flesh e*^*3756*^ (also known as *yellow flesh, locus r*^*3756*^) allele is an EMS mutant with a premature stop codon in *PSY1* leading to a yellow fruit phenotype^39^. The *bicolor*^*cc383*^ allele (also known as *yellow flesh Bicolor, locus r*^*Bi*^) is a mutant with a 3.7 Kb deletion in the promoter of *PSY1* leading to a yellow–red moulted fruit phenotype (Fig. 1a). In order to monitor the CRISPR-Cas-induced mutations throughout plant development, starting from fertilization, we produced transgenic *yellow flesh e*^*3756*^ lines, expressing 35S:Cas9 and transgenic *bicolor*^*cc383*^ lines expressing a *PSY1* single guide RNA (u6-26:Ps#1-sgRNA). This u6-26:Ps#1-sgRNA was designed to induce a DNA DSB between the *bicolor*^*cc383*^ and *yellow flesh e*^*3756*^ mutations, on both alleles (Fig. 1a). It is located 1,086 bp downstream from the deletion in *bicolor*^*cc383*^ and 556 bp upstream from the *yellow flesh e*^*3756*^ mutation. A cross between *yellow flesh e*^*3756*^ 35S:Cas9 and *bicolor*^*cc383*^ u6-26:Ps#1-sgRNA is expected to yield F_1_ plants with the dominant *bicolor*^*cc383*^ fruit phenotype. The same is expected for control plants that do not express either 35S:Cas9 or u6-26:Ps#1-sgRNA. Deviations from this phenotype in plants expressing both Cas9 and Ps#1-sgRNA are expected due to the induction of a DSB on one or both alleles followed by DNA repair. A NHEJ repair of the *bicolor*^*cc383*^ allele, or of both alleles, should yield a yellow fruit phenotype (sectors or whole fruit). The outcome of DSB repair by HR based mechanisms (inter-homologues crossover or non-crossover events), could be a red fruit in case of an HR event that occurred early in development, or fruits with red spots or sectors in a yellow or bicolour background in case of late events (Fig. 1a, Supplementary Fig. 1).

Upon DSB induction, a population of 50 *yellow flesh e*^*3756*^ 35S:Cas9 × *bicolor*^*cc383*^ u6-26:Ps#1-sgRNA F_1_ plants, gave bicolour, yellow and yellow with red spots fruits. The distribution of fruit phenotypes varied when we used *yellow flesh e*^*3756*^ 35S:Cas9 transgenic lines originating from independent transformation events (as shown in Fig. 1b). As expected, in the absence of DSB induction, the control population of six *yellow flesh e*^*3756*^ × *bicolor*^*cc383*^ F_1_ plants, showed only bicolour fruits (Fig. 1b). One of the advantages of our fruit colour assay is its ability to predict the inheritance of repair products in the next generation. Indeed, we expected that most of the F_2_ seeds extracted from totally yellow fruits would give rise to a germinally transmitted mutation. To confirm that yellow fruits are indicative of NHEJ germinal events, we grew F_2_ plants derived from yellow fruits. Using allele-specific PCR amplification of the *yellow flesh e*^*3756*^ and *bicolor*^*cc383*^ alleles and sequencing of the PCR products, we showed that in all cases tested seeds from yellow fruits yielded seeds carrying a germinally transmitted mutation at the DSB site of the *bicolor*^*cc383*^ allele (Supplementary Fig. 2). Some of the progeny from yellow fruits also showed mutation in the *yellow flesh e*^*3756*^ allele (Supplementary Fig. 2). Although we found many yellow fruits with small red sectors (Fig. 1b), we did not detect any fully red fruit among the F_1_ plants. In addition, we grew a population of 400 F_2_ plants derived from fruits with red spots, suggestive of somatic HR, but we did not detect any fully red fruit that would indicate germinally transmitted HR events.

### CRISPR DNA DSB repair via both somatic NHEJ and HR

In order to identify, characterize and quantify somatic NHEJ events in F_1_, we used 22 F_1_ plants of *yellow flesh e*^*3756*^ 35S:Cas9 × *bicolor*^*cc383*^ u6-26:Ps#1-sgRNA and 2 F_1_ plants of *yellow flesh e*^*3756*^ × *bicolor*^*cc383*^as control. We collected four leaves from different branches of the plants. Then, we extracted their DNA, amplified the region flanking the induced DSB of both alleles by PCR and sequenced the resulting products using high-throughput sequencing Illumina HiSeq 2500 platform. Out of 250,000–850,000 reads per plant (PCR sample), an average of 88% of the reads of *yellow flesh e*^*3756*^ 35S:Cas9 × *bicolor*^*cc383*^ u6-26:Ps#1-sgRNA plants contained a mutation at the CRISPR DSB site, while only 2% of the illumina reads of *yellow flesh e*^*3756*^ × *bicolor*^*cc383*^ plants deviated from the WT sequence, presumably due to PCR and sequencing errors (Fig. 1c). The high rate of CRISPR-Cas DSB induction in the system, lead to a broad spectrum of mutations as a result of different NHEJ repair events. In addition, we found that some NHEJ signatures, such as the 4 bp CTTG deletion, were preferred over others at this locus (Fig. 1c, Supplementary Fig. 3).

For the measurement of HR repair, we designed an inverse PCR method that allowed the sequencing of the two allele-specific mutations which are 1.6 Kb apart (*yellow flesh e*^*3756*^ and *bicolor*^*cc383*^), enabled to distinguish parental from recombinant molecules (Fig. 1d, Supplementary Fig. 4) and minimized the formation of false positive PCR products. The same DNA samples used for the somatic NHEJ sequencing (Fig. 1c) were used for the inverse PCR. In addition, we cloned two synthetic positive controls (recombinant-like clones) that were also treated by the same inverse PCR method. The products of the inverse PCR from each reaction (as shown in Fig. 1d) were sequenced by Illumina HiSeq 2500 paired-end sequencing. In this assay we got 5,000–50,000 reads per plant. The negative controls of *yellow flesh e*^*3756*^ × *bicolor*^*cc383*^ only showed the parental alleles in the absence of DSB induction, while the positive synthetic control showed the recombinant alleles (Fig. 1e). Most F_1_ plants of *yellow flesh e*^*3756*^ 35S:Cas9 × *bicolor*^*cc383*^ u6-26:Ps#1-sgRNA showed only the parental alleles but some of them showed one of the recombinant alleles, suggesting somatic HR based repair.

### Allele-specific DSB induction

**Figure 2:**
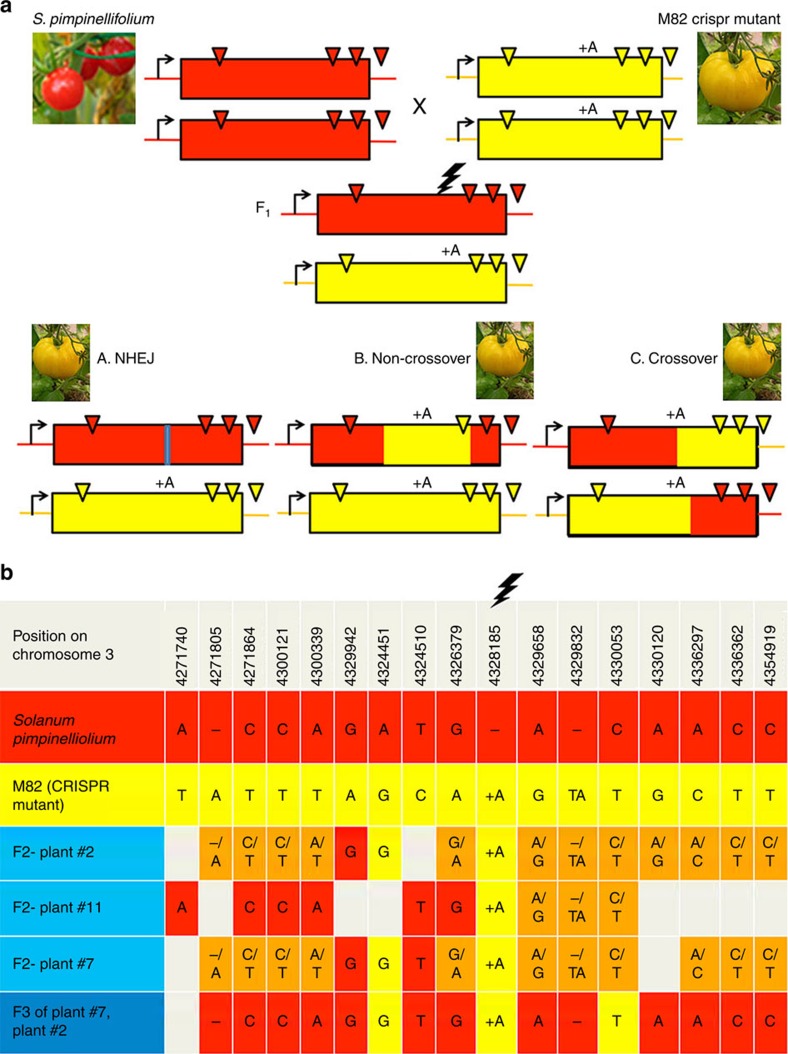
Tomato SNPs assay for crossover and non-crossover events. (**a**) An homozygote M82 CRISPR mutant (+A, +A) expressing 35S:Cas9 and u6-26:Ps#2-sgRNA was crossed with *S. pimpinellifolium*^*LA1578*^. The F_1_ is expected to give red fruits without DNA DSB and yellow fruit in case that the break was repaired by NHEJ, non-crossover or crossover. The SNPs pattern is allowing differentiating between repair mechanisms. Triangles are for SNPs; lightning mark the DSB site; blue line is for NHEJ indels. (**b**) Analysis of DNA DSB flanking markers in F_2_ and F_3_ plants. Red- homozygote for *S. pimpinellifolium*^*LA1578*^ SNPs; yellow- homozygote for M82 SNPs (including the +A CRISPR-Cas9 mutant); orange- heterozygote; empty cells are for missing data; lightning- DSB site.

The above cross between *yellow flesh e*^*3756*^ × *bicolor*^*cc383*^ did not provide enough SNPs to analyse in detail the HR repair products. Moreover, it did not enable to perform an allele-specific break, which is needed to perform a precise experiment where it is possible to distinguish between the broken chromosome and the repair template. Therefore, we designed a new DSB repair assay that provides several SNPs around the break site as well as in distal regions, through the use of *Solanum pimpinellifolium*^*LA1578*^, a wild tomato accession with small red fruits and a sequenced genome showing the presence and location of multiple SNPs compared to *Solanum lycopersicum*, the edible tomato. In order to ensure allele-specific break, we mutated an allele in the *S. lycopersicum* M82 cultivar background, that is immune to u6-26:Ps#2-sgRNA. For that purpose, we transformed the red fruits cv. M82 with 35S:Cas9 and u6-26:Ps#2-sgRNA. Then, we selected for yellow fruits in T_0_ and grew their T_1_ seeds. From this T_1_ population, we isolated a homozygote plant with an adenine insertion (+A) at the CRISPR-Cas9 DSB site and crossed it with the wild tomato accession. In this assay, the *S. pimpinellifolium*^*LA1578*^ is the only target for DNA DSB due to the +A insertion, in the M82 *psy1* allele, that disrupts the protospacer adjacent motif (PAM) and prevents Cas9 cleavage. The +A mutation of M82 allele is recessive and therefore F_1_ plants are expected to have small red fruits. DSB repair in *PSY1* by NHEJ, or HR (crossover or non-crossover) leads to yellow fruits or red fruits with yellow sectors, depending on the developmental fruit stage when the repair occurred. NHEJ repair events are expected to leave small indels at the DSB site, while crossover and non-crossover events can be identified by the difference in SNPs patterns on both sides of the DNA DSB (Fig. 2a).

For the analysis of somatic DSB repair, we PCR-amplified and sequenced by Illumina HiSeq 2500 paired-end sequencing the DSB DNA area from leaves of both parents (M82 35S:Cas9, U6-26:gRNA, +A homozygote and *S. pimpinellifolium*^*LA1578*^) and from five F_1_ plants (Supplementary Fig. 5). This sequencing yield was 600,000–900,000 reads per plant. We show that the M82 *psy1*^+A^ allele was immune to DSB induction, with virtually no DSB footprints in the M82 (M82 35S:Cas9 and u6-26:Ps#2-sgRNA, +A homozygote) parent, supporting the designed allele-specificity of the gRNA (Supplementary Fig. 5). In addition, at least 50% of the reads gave the +A insertion while the *S. pimpinellifolium* allele was mutated (red colour in the pie chart of Supplementary Fig. 5). We found that only 7–18% of the F_1_ plants reads were WT and the rest gave various indel patterns (Supplementary Fig. 5). To estimate the rate of germinal events, we documented the fruit colours on different branches and sequenced the fruit pericarp tissue by Illumina (Supplementary Fig. 6). With this assay, the fully yellow fruits might contain seeds that are germinal events of repair via NHEJ or HR (Fig. 2a). Moreover, crossover or non-crossover events should give +A, +A homozygote plant as the repair for template is the M82 *psy1*^+A^ allele (Fig. 2a). In one of the F_1_ Plants, we found yellow fruits that showed high +A, +A content by illumina and Sanger sequencing (Supplementary Figs 6 and 7). We grew the F_2_ progeny of this F_1_ plant and sequenced them by the Sanger method. The sequencing revealed F_2_ plants with SNPs patterns corresponding to germinal HR events (Fig. 2b). Plants #2 and #7 look like clear cases of non-crossover, both with conversion tracts of at least 5 Kb. These plants originate from different F_1_ fruits on the same F_1_ plant and therefore might correspond to the same recombination event. Plant #11 looks like a case of crossover (Fig. 2b), however, the analysis of flanking markers (Indels and SNPs), more than 20 kb away from both sides of the DSB site in plant #11 could not be performed due to plant death, therefore, we refer to this case as a putative crossover. To identify homozygote gene conversion products, and to better characterize the borders of the conversion tract, we sequenced F_3_ plants from the progeny of plant F_2_ #7. One of the F_3_ progeny of F_2_ #7 (Shown at the bottom of Fig. 2b) is an homozygote product of gene conversion repair with a confirmed conversion tract of 5–6 kb length. Interestingly, the conversion tract seems interrupted with SNPs from *S. pimpinellifolium* within M82 (see discussion for possible mechanisms).

### Quantification of the rate of allele-dependent repair

**Figure 3:**
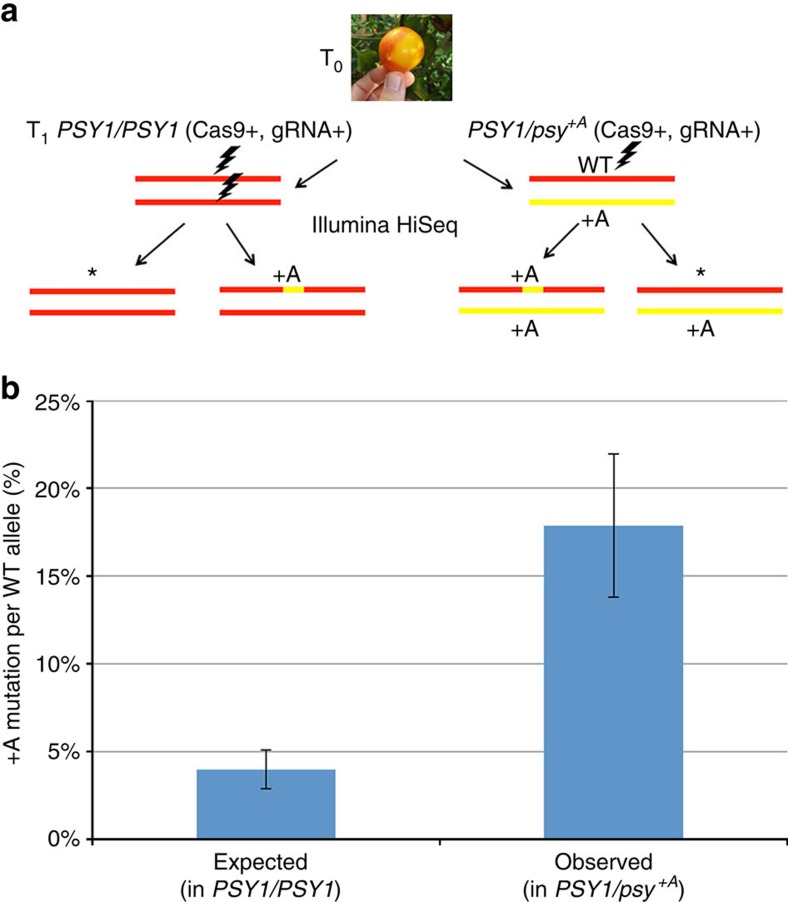
Quantification of allele-dependent repair. (**a**) Two plant populations were grown, both in the M82 background: one homozygote for *PSY1/PSY1* and the other heterozygote for the *PSY1/psy*^*+A*^ genotype. Progeny of these plants could give a +A SNP at the site of the break (lightning) or any other mutation (*). DNA was extracted from leaves of 4-week-old plants of both populations and sequenced by illumina. In the *PSY1/PSY1* plants, both alleles can be targeted, while in the *PSY1/psy*^*+A*^ plants, only the WT *PSY1* allele is targeted. (**b**) The per cent of +A mutation per WT allele in *PSY1/PSY1* plants served as expected value for allele-independent +A mutation. It was calculated by the following equations: Expected=(%(+A reads)_*T*=4 weeks (wt, wt)_)/2. To estimate the observed occurrence of the +A mutation when the second allele has a +A mutation (in M82-WT *PSY1*/ M82 *psy1*^+A^ heterozygote plants) as shown in panel A, we used the equation: Observed=%(+A reads)_*T*=4weeks, (wt, +A)_−50%. The bars correspond to the s.e. for 22 *PSY1/PSY1* plants and 14 *PSY1/psy*^*+A*^ plants. The difference between the means was significant (*P*=0.009, Wilcoxon rank sum test, *n*_homozygote_=22, *n*_hetrozygote_=14).

Thanks to the system we developed of allele-specific DSB induction, we were able to test allele-dependent repair, a signature of HR between homologues. Induction of DNA DSB on the *S. pimpinellifolium*^*LA1578*^ allele showed the +A signature, similar to the M82 *psy1*^+A^ allele, at the broken site in many of the fruits and leaves we sequenced (Supplementary Figs 6 and 7). This excess in +A repair might be due to preferred NHEJ repair pattern or to allele-dependent repair mediated by HR. To distinguish between these two possibilities, we grew several plants of the M82 cultivar, all of them offspring of the same 35S:Cas9 u6-26:Ps#2-sgRNA. In this population, 22 plants were initially homozygote for the M82-WT allele of *PSY1*, while 14 plants were initially heterozygote M82-WT *PSY1*/ M82 *psy1*^+A^. We grew the plants to the age of 4 weeks and collected four leaves from each of them. Then, we amplified DNA around the DSB by PCR and sequenced the PCR products with the Illumina HiSeq 2500 platform. For each plant, we calculated the percentage of each indel out of the total number of reads. In case that the +A mutation occurs independently in each chromosome, there should be twice more reads with new +A mutations in the WT (which has two potential targets) than in the heterozygote where only one target is available (Fig. 3a). It is possible though that in the WT a first mutation, repaired by NHEJ would generate the +A footprint. Following this event, a second mutation at the homologous target could be repaired by HR using the newly generated +A allele in the homologue. We chose to ignore this possibility, and to assume that all repair events in the WT occur by NHEJ in order to establish the most stringent threshold as control in our estimation of inter-homologue HR repair. To measure the expected allele-independent +A NHEJ footprint per chromosome, we used the 22 plants of the WT homozygote and calculated the per cent of +A reads divided by two to obtain the value of the occurrence of the +A mutation per allele. The following equation was used: Expected=(%(+A reads)_*T*=4 weeks (wt, wt)_)/2. The occurrence of a new +A mutation in the WT allele, when the second allele contains the +A mutation (in M82-WT *PSY1*/M82 *psy1*^+A^ heterozygote plants) is calculated by taking the percentage of +A reads in the M82-WT *PSY1*/M82 *psy1*^+A^ plants and deducing 50% (the initial per cent of reads originating from the M82 *psy1*^+A^ allele). We used the following equation for the observed rate of +A mutation in heterozygote M82-WT *PSY1*/M82 *psy1*^+A^ plants: Observed=%(+A reads)_*T*=4 weeks, (wt, +A)_–50%. When we compared the expected to the observed +A footprint, we found a significantly higher than expected rate of novel +A mutations in the heterozygote population (*P*=0.009, Wilcoxon rank sum test, *n*_homozygote_=22, *n*_hetrozygote_=14). Considering that the two populations are isogenic, this suggests that the repair at the site of DSB is dependent on the sequence of its homologous allele (Fig. 3b). The average allele-independent frequency of a +A footprint was 4% per allele in the M82-WT (under the assumption that all repair in the WT occurred by NHEJ), while the average +A footprint frequency in the M82-WT *PSY1* allele in M82-WT *PSY1*/M82 *psy1*^+A^ heterozygote was 18% (Fig. 3b). This suggests that 18%–4%=∼14% of the DSB repair events are allele-dependent and the rest occurs via NHEJ in an allele-independent manner. Note that probably, some of the repair events in the WT occurred via inter-homologue HR, therefore the rate of allele-dependent repair would be even higher than the 14% estimate.

## Discussion

Earlier studies on somatic DSB-induced HR repair were done mostly with transgenic assays for intrachromosomal recombination^27^,^28^ or inter chromatids unequal crossover^29^ or inter-homologues recombination^40^. The assays developed here were carried in an endogenous genomic context where the repair template origin could be tracked on the homologous chromosome. We present a series of evidence showing that targeted DSBs can be repaired via somatic HR using an homologous chromosome as the template. In addition, we demonstrated that some of these repair events can be transmitted germinally to the next generations. In one set of crosses, we showed that we can recover the WT allele through intragenic recombination between two defective *psy1* parental alleles (*bicolor*^*cc383*^ and *yellow flesh e*^*3756*^), an event seen as red spots (Fig. 1a) and characterized through sequence analysis (Fig. 1e). In this cross, we did not recover fruits that were fully red, that would correspond to early germinal events. This might be due to the genomic context of the large deletion in the bicolor allele, or alternatively the ‘cured' recombinant WT allele underwent a second round of NHEJ during development (the target site was not destroyed during HR), that would generate a loss of function (yellow) allele via NHEJ. Considering the high efficiency of NHEJ, this is a plausible scenario. In addition, in an assay of allele-specific DSB induction in a *S. pimpinellifolium* × *S. lycopersicum* F_1_ hybrid, we found three cases of HR-dependent repair that were germinally transmitted to the F_2_ and F_3_ generations. Two cases corresponded to non-crossover events with conversion tracts of 5–6 Kb (Fig. 2b). The third case (F_2_ plant #11) is a germinal HR event that might be either a crossover event or a non-crossover event—this could not be demonstrated due to the plant death. Finally, trying to quantify the ratio of HR versus NHEJ, we designed a sgRNA for allele-specific DSB induction in the *S. lycopersicum* background. This experimental set-up enabled to measure an excess of repair footprints originating from the homologous allele compared to expectation, suggesting that out of all the detectable DSB repair events at least 14% are allele-dependent and the rest is non-homologous.

It is interesting to compare HR-mediated repair in somatic versus meiotic cells. Overall little is known on inter-homologues recombination in somatic tissues probably owing to the low frequency of such events, to the lack of phenotypic markers and to the difficulty to retrieve germinal events. We could not detect any red sector in the absence of DSBs, and the presence of intragenic recombinant molecules was null or negligible. This is consistent with earlier studies in tobacco showing that the occurrence of somatic HR is very low in the absence of DSB induction for both reciprocal and non-reciprocal HR events^41^,^42^. The low rates of somatic HR between homologous chromosomes might be indicative of bottlenecks such as absence of the HR machinery found in meiosis that controls homologues pairing, synaptonemal complex formation and so on. Our results, showing a relatively high rate of HR-repair based on both case-studies and on quantitative assessments, indicate that DSBs are a major bottleneck, inducing somatic HR from 0 (in the absence of breaks) to ∼14% per allele (allele-dependent repair measured in Fig. 3b) and that DSB-induced HR between homologues can occur in the absence of the meiotic HR machinery.

The rate of HR DSBs repair that we report here (of 14% per allele or more) is apparently higher than that reported during meiosis. Indeed, only a small fraction of meiotic breaks (∼3–5%) evolves into crossovers^12^,^43^ and a similar fraction is repaired as non-crossover^43^,^44^. However, meiotic non-crossover rates might be underestimated because such events may go un-noticed if they happen between sister chromatids or between homologous chromosomes with a short conversion tract that does not include SNPs as suggested by Drouaud *et al*.^44^. Moreover, the somatic HR DSB repair we report here could have happened during several cellular divisions during development (starting from the zygote) until error-prone repair destroys the target, while in meiotic cells, there is only one cell division when Spo11 induces hundreds of breaks in the genome. Therefore, it is premature to make meaningful statements on the difference in HR rates between somatic and meiotic recombination. Likewise, we presented several evidence on the occurrence of DSB-mediated HR repair, however in most assays we could not distinguish between crossover versus non-crossover repair mechanisms. The analysis of three germinal events in a polymorphic background enabled to perform this distinction but the sample (of two conversions and one putative crossover) is too small to draw conclusions.

We found a rather long (∼5 kb) conversion tract where homozygous *S. pimpinellifolium* SNPs at positions 4324510, 4326379, 4329658 and 4329832 appeared within the M82 homozygous patch at the boundaries of the tract (Fig. 2b, F3 plant). It might be these SNPs were part of a large heteroduplex region in Holliday junctions and that mismatch repair at these positions generated a discontinuous conversion tract^45^. Alternatively, it might be that such chimeric tract was formed via the Synthesis-dependent-Strand-Annealing pathway and that multiple template switches happened during DNA synthesis. Such examples of multiple template switches were shown in plants^21^,^30^ and in other species^46^. Earlier reports on meiotic conversion tracts showed mean values of 552 bp ref. 44. This length difference might reflect a difference between species (tomato versus *Arabidopsis*) or between meiotic and somatic cells or it might be due to the experimental set-up (Spo11 versus Cas9 DSB induction).

Finally, we showed that it is possible to induce germinally transmitted targeted HR between endogenous homologous chromosomes in somatic cells. It remains to be shown if and how efficiently targeted HR can occur during meiosis.

Our results suggest that custom-designed nucleases, such as CRISPR-Cas, can be used for precise reshuffling of chromosomal segments between homologous chromosomes in somatic cells. For example, it might be possible to transfer a disease resistance gene from a wild relative to the crop, without a long process of backcrossing which not only takes several generations in order to achieve isogenic lines, but also drags large segments of undesirable DNA flanking the desirable gene. In other words, we showed that somatic HR can be used for allelic replacement. Another potential application of DSB-induced somatic HR is ‘targeted crossover', that is, the reciprocal exchange of large chromosomal segments at a precise site. Crossing over between homologous chromosomes during meiosis is the engine of breeding in sexually reproducing crops, but it takes place at uncontrolled and unpredictable sites in the genome. Obtaining rare recombination events—such as shown here for intra-allelic crossover—is a major hindrance in most breeding programs. We propose to exploit targeted somatic HR to segregate between undesirable genetic linkages. Somatic crossover does occur in plants^27^,^28^,^29^,^30^,^31^,^32^, and can even reach high levels in some mutants^47^ suggesting that the inter-homologue crossover machinery is available in somatic tissues and that targeted crossover is feasible. Interestingly, even-though the meiotic crossover machinery has been optimized during evolution, the targeted induction of a given DSB during meiosis would have to compete with the hundreds of naturally occurring other breaks as a substrate for crossover and counter to intuition, might turn out to be less efficient than somatic HR for targeted crossover induction.

## Methods

### Plant material

All tomato plants were grown in greenhouse conditions with temperature ranging between 18 and 25 °C. The tomato (*S. lycopersicum*) mutant line of *yellow flesh e*^*3756*^, *Bicolor*^*cc383*^, M82 and *S. pimpinellifolium*^*LA1578*^ were kindly provided by the labs of Prof. Joseph Hirschberg and Prof. Daniel Zamir at the Hebrew university of Jerusalem (http://zamir.sgn.cornell.edu/mutants/)^39^.

### Plasmids and plant transformation

We used the 35S:Cas9 and u6-26:sgRNA constructs^48^ and the u6-26 promoter^49^,^50^ which was synthesized by IDT (Integretad DNA Technologies) based on *Arabidopsis thaliana* sequence. The primers used for construction of ps#1-sgRNA and ps#2f targets are specified in Supplementary Table 1. All tomato lines were transformed by *Agrobacterium tumefaciens* GV3101 using cotyledon transformation^51^.

### Inverse PCR for HR detection

DNA samples for the Inverse PCR assay were extracted using a DNA purification kit (MACHEREY-NAGEL). For each plant 300 ng of DNA from leaves sample or control plasmid were treated separately: first, they were incubated over night with 10 × FD buffer, ApaLI (ThermoFisher scientific) and HindIII-HF (New England BioLabs). After 20 min of 80 °C inactivation, 150 ng of the digested fragments were blunted with T4 DNA polymerase (New England BioLabs) for 2 hs at room temperature. The T4 DNA polymerase was inactivated at 75 °C for 10 min and the linear DNA was self-ligated with Quick T4 DNA ligase (New England BioLabs) for 30 min at room temperature. Control plasmids were diluted 1:10,000 with DDW and mixed together for mimicking ‘heterozygosity'. Then all samples were amplified by 18 cycles of PCR with Phusion High-Fidelity DNA polymerase (New England BioLabs) (for primers see primers list). The primers for this assay were designed for allele-specific amplification. Samples were pooled and sequenced by high-throughput sequencing.

For the cloning of the synthetic crossover-control plasmids, two PCR fragments were amplified from *yellow flesh e*^*3756*^ and *Bicolor*^*cc383*^ DNA samples using Phusion. High-Fidelity DNA polymerase (New England BioLabs) (for primers sequence see primers list) and then cloned using the GoldenBraid cloning system^52^ (https://gbcloning.upv.es/). First, each of the four amplicons was cloned into the pUPD plasmid. Then pUPD2 plasmid with ‘ps' segment from *yellow flesh e*^*3756*^ was cloned with pUPD2 plasmid with ‘y1' segment from *Bicolor*^*cc383*^ into pDGB3_alpha1 plasmid. In parallel, pUPD2 plasmid with ‘ps' segment from *Bicolor*^*cc383*^ was cloned with pUPD2 plasmid with ‘y1' segment from *yellow flesh e*^*3756*^ into pDGB3_alpha1 plasmid. These two ‘synthetic allele' plasmids were pooled together and were subjected to inverse PCR and sequencing. For the specific sequence of synthetic plasmids see R1 and R2 in Supplementary Note 1.

### DNA amplification and sequencing

DNA samples for high-throughput sequencing were amplified using Phusion. High-Fidelity DNA polymerase (New England BioLabs) and 18 PCR cycles (for specific primers of each experiment see primers list). Libraries were prepared as Blecher-Gonen *et al*.^53^. High-throughput Sequencing was performed at the G-INCPM unit at the Weizmann Institute of Science with the Illumina HiSeq 2500 platform for 2 × 125 paired-end reads.

DNA samples for Sanger sequencing were amplified using REDTaq (SIGMA-ALDRICH) with 35 PCR cycles (for primers see Supplementary Table 1). Then cleaned with Exonuclease I and Shrimp Alkaline Phosphatase (rSAP) (New England BioLabs). Sequencing was performed at the Biological services unit at the Weizmann Institute of Science with Applied Biosystems 3730 DNA Analyzer.

### Data availability

Sequencing data associated with this study has been deposited in the NCBI Sequence Read Archive under BioProject accession PRJNA380032. The authors declare that all data supporting the findings of this study are available within the manuscript and its supplementary files or are available from the corresponding author upon request.

## Additional information

**How to cite this article:** Hayut, S. F. *et al*. Targeted recombination between homologous chromosomes for precise breeding in tomato. *Nat. Commun.* **8,** 15605 doi: 10.1038/ncomms15605 (2017).

**Publisher's note:** Springer Nature remains neutral with regard to jurisdictional claims in published maps and institutional affiliations.

## Supplementary Material

Supplementary Information Supplementary Figures, Supplementary Table and Supplementary Note
