# Supplementary material for: Targeted recombination between homologous chromosomes for precise breeding in tomato
Source: Nat Commun. 2017 May 26;8:15605. doi: 10.1038/ncomms15605 (PMC5458649; doi:10.1038/ncomms15605)
Supplement: Supplementary Information — Supplementary Figures, Supplementary Table and Supplementary Note [file ncomms15605-s1.pdf]

Supplementary Information:

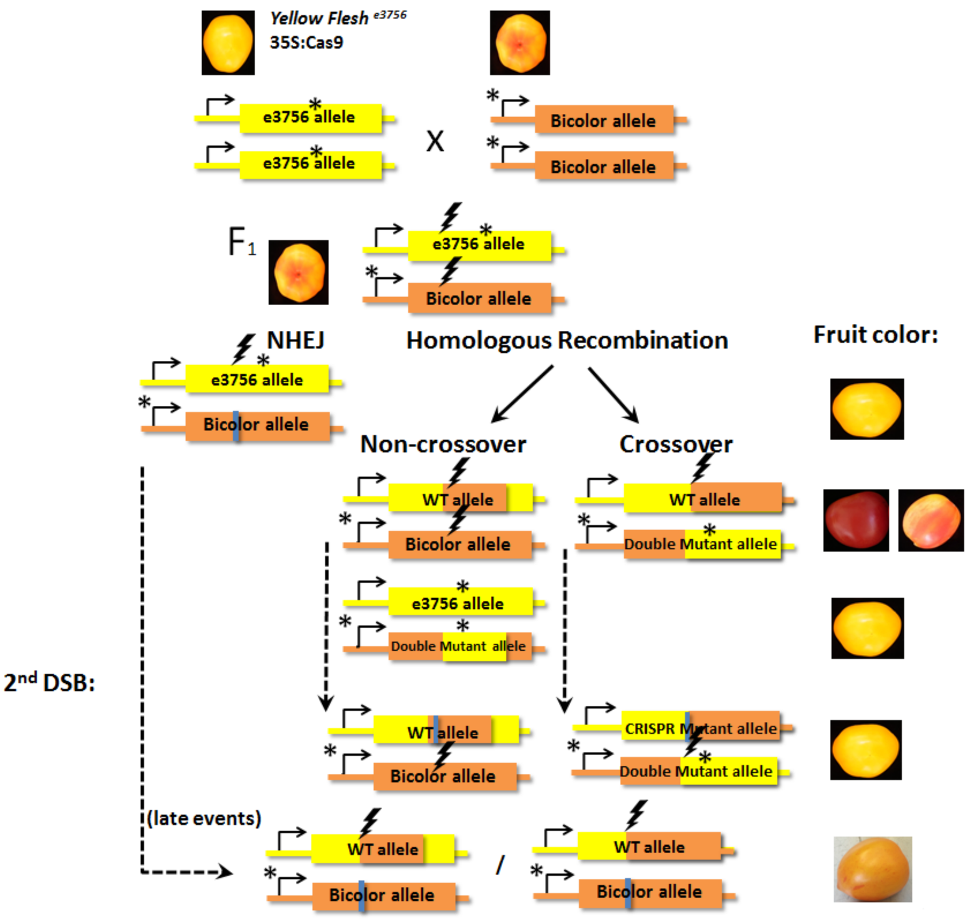

Supplementary Figure 1 - Tomato fruit color assay and molecular analysis for possible outcomes of DNA double strand break (DSB) repair events (Extended Fig 1A). The cross of *yellow flesh*^{e3756} 35S:Cas9 and *bicolor*^{cc383} u6-26:Ps#1-sgRNA is expected to give F_1 plants with a pale Bicolor fruit phenotype. F_1 plants expressing both Cas9 and gRNA were selected. The gRNA was designed for DSB induction (shown as black lightning) in both alleles between the *yellow flesh*^{e3756} and *bicolor*^{cc383} mutations (*) 1086 bp from the deletion in bicolor and 556 bp from the *yellow flesh*^{e3756} mutation. In case of NHEJ repair of the *bicolor*^{cc383} allele, or of both alleles, fruit color is expected to be yellow following error-prone repair leaving indel footprints (blue line). In some of the cases of non-crossover or crossover, fruit color is expected to be red or bicolor with red spots in case of late event. Note that red fruits are shown as expected recombination products but were not found. Rather, fruits with red spots in a yellow or *bicolor* background were found and are shown (together with various putative products of HR-induced repair). In case that only one allele experienced DNA DSB, the second DNA DSB event (in dashed arrow) may be repaired independently. For example, NHEJ in the *bicolor*^{cc383} allele can

be followed by a late event of HR-mediated DSB repair of *yellow flesh*^{e3756}. This would give rise to yellow fruits with red spots—a phenotype that was observed. Generally, when repair via HR restored the WT allele, the CRISPR target site is still available for subsequent DSB induction. Therefore, yellow fruits may be the result of different repair mechanisms. Additional scenarios can be obtained depending on both timing and mechanism of DSB repair in each allele.

a

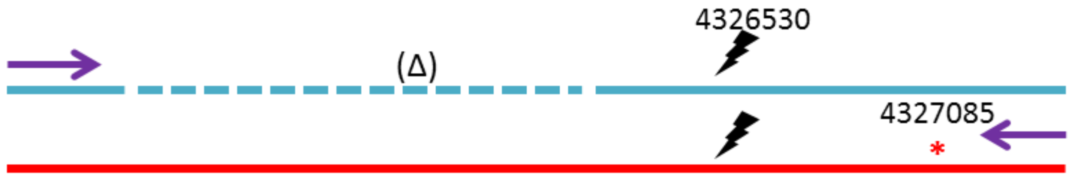

b

F1 plant	Fruit number	F2 plant	Cas9	gRNA	*Yellow flesh* allele (3756)	*Bicolor* allele (Bic)
1	1	11	-	+		Bic/4bp del
16	1	1	+	+	3756/4bp del	T insertion
16	1	2	-	-	3756	T insertion
16	2	3	+	-	3756	T insertion
16	2	4	-	+	3756	T insertion
16	3	1	+	-	3756	T insertion
16	3	2	+	-		T insertion
18	1	1	+	+		4bp del
18	1	4	+	+	T insertion	4bp del
18	2	2	+	+		4bp del
45	1	1	+	-	3756	G insertion
45	1	6	+	-	3756	G insertion
45	3	1	+	+	3756	G insertion
45	3	4	+	-	3756	G insertion
45	3	5	+	-	3756	G insertion

c

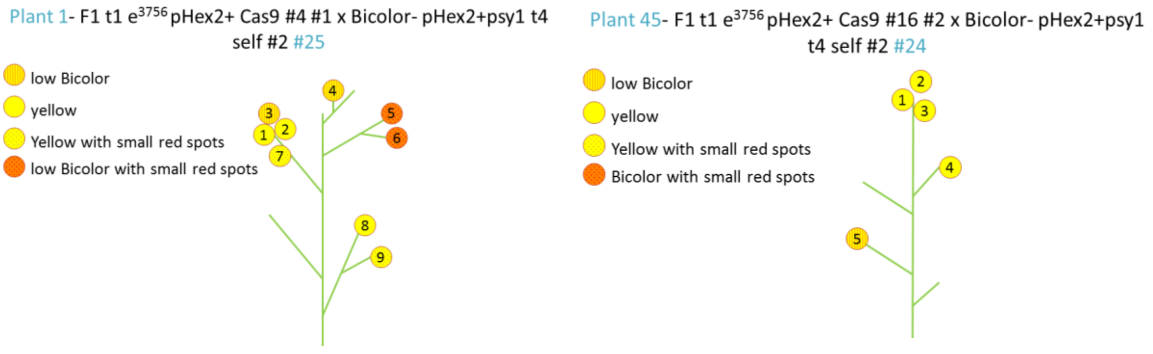

Supplementary Figure 2 - NHEJ germinal events derived from yellow fruits. A. *bicolor*^{cc383} allele in blue, *yellow flesh e*^{3756} in red, purple arrows represents primers that flanking the *bicolor* mutation and amplifying 2 PCR products in different sizes. Each product extracted from gel and then sequenced by Sanger. B. Allele specific PCR products of F_2 plants derived from yellow fruits of F_1 *yellow flesh e*^{3756} 35S:Cas9 x *bicolor*^{cc383} u6-26:Ps#1-sgRNA were sequenced by Sanger sequencing. All the plants

contained indels at the *bicolor* allele. 2 out of 11 contained indels in the *yellow flesh* allele as well. In some cases, the *yellow flesh* allele was not amplified due to homozygosity for *bicolor*. For some of the alleles we saw two bicolor alleles: the *bicolor*^{cc383} allele and a *bicolor*^{cc383} allele with a 4bp deletion mutation (e.g. Plant F2#11 derived from fruit#1 in F1). This apparent discrepancy between the F1 yellow fruit color and this *bicolor* genotype can be explained by independent events that occurred during the development of F1 flowers & fruits (different mutations in the maternal pericarp and ovule versus the non-mutated pollen cells as in F1 plant#1 fruit#1). C. In support of this, the fruits on F2 plant #11 were all *bicolor*. Moreover, the schematic map of F1 plant#1 shows that within some fruit clusters (as in F1 plant #1 fruit cluster 1,2,3,7) the DSB repair event seems to have occurred late, resulting in different fruit colors on the same fruit cluster. While in other clusters (as in F1 plant #16 fruit cluster 1,2,3) the DSB event seems to be early and characterized by uniform fruit phenotype and genotype. The illumina Hiseq result in Fig S3 are also supports early G insertion in F1 plant #45. . In plant F2#1 derived from F1#16 we saw 3 different alleles. This can be explained by the presence of both Cas9 and gRNA in this plant.

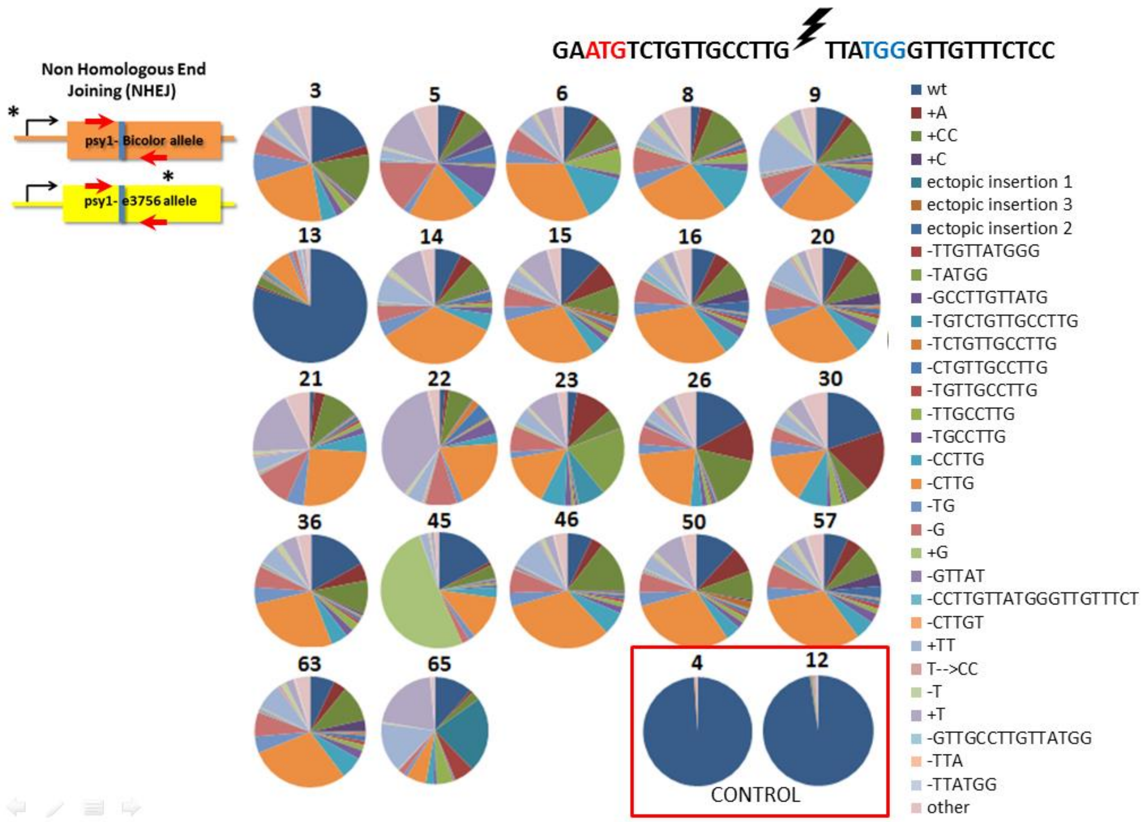

Supplementary Figure 3 - NHEJ repair in somatic cells. NHEJ footprints distribution in individual F_1 plants and in control plants (*yellow flesh* e^{3756} x *bicolor*^{cc383}) obtained by sequencing of PCR products amplified around the CRISPR-Cas9 induced DSB (lightning) with primers shown as red arrows. Each pie represents the total illumina Hiseq reads for single plant (250,000-850,000 reads per plant).

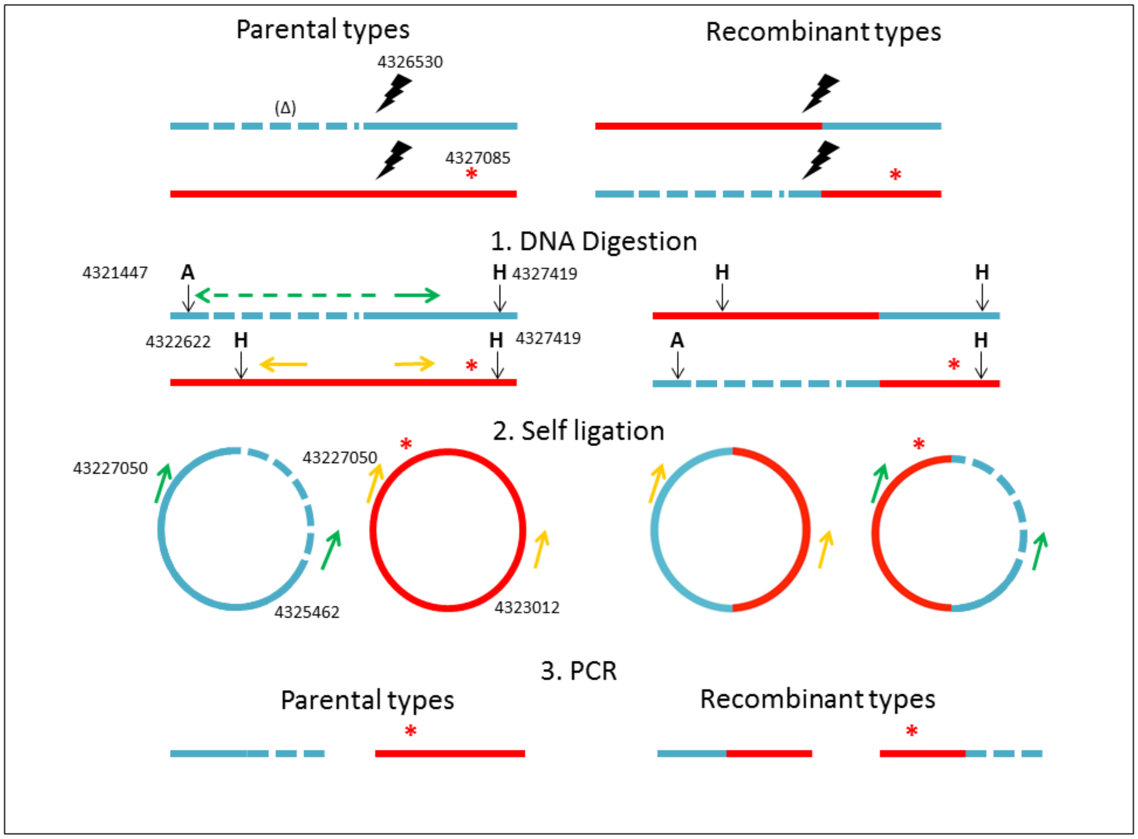

Supplementary Figure 4 - Inverse PCR detailed scheme for identification of recombinant DNA fragments. The specific coordinates of *bicolor*^{cc383} and *yellow flesh e*^{3756} mutation and of DNA DSB site on tomato chromosome 3 are listed above the *bicolor*^{cc383} and *yellow flesh e*^{3756} parental types alleles. (1) DNA from separate leaves samples was first digested with ApaI(A) and HindIII(H) and then blunted. The coordinates of ApaI(A) and HindIII(H) are listed next to the relevant restriction sites. (2) Each sample was self-ligated. (3) Each sample was amplified by two different primer sets shown as green and yellow arrows. Primers coordinates are listed next to each primer; for primer sequences see primers list). The reverse primers are designed specifically for each allele: The green dashed arrow represents a bicolor-specific primer that spans both sides of the deletion (hence the dashed line), The Yellow reverse primer is specific to the *yellow flesh e*^{3756} allele because it is inside the region that corresponds to the deletion in bicolor. Blue- *Bicolor* allele; red- *Yellow flesh* allele; Dashed blue line- *Bicolor* deletion, *- *Yellow flesh* mutation, lightning- DSB site.

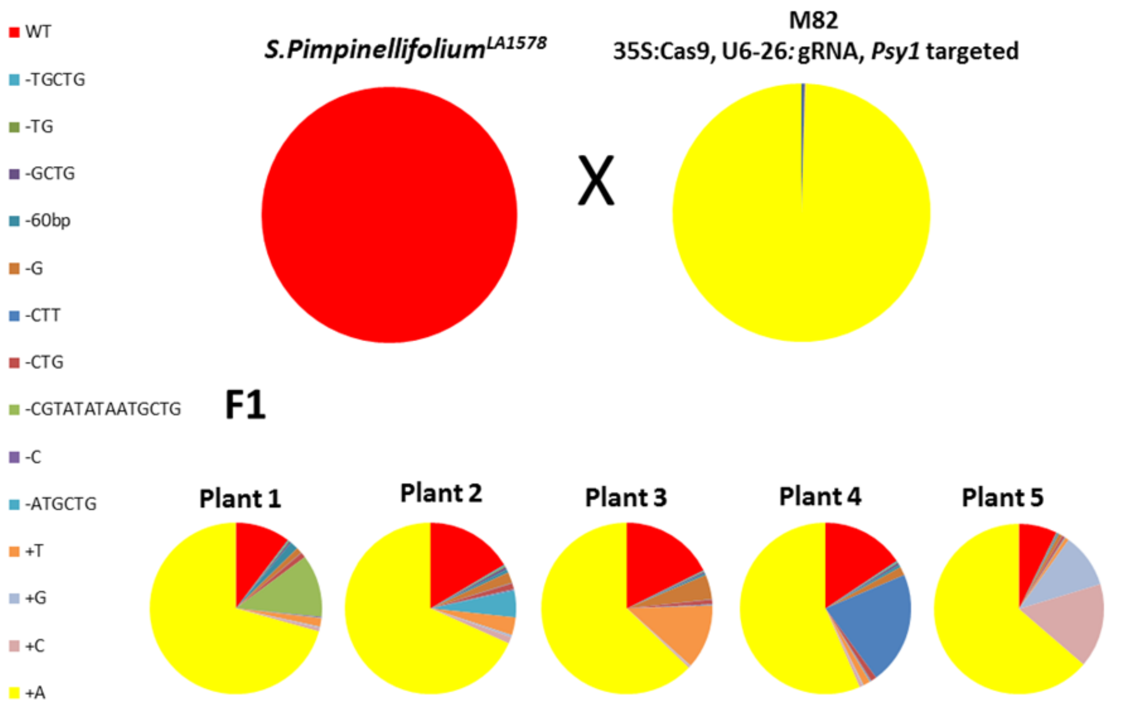

Supplementary Figure 5 - Tomato SNPs assay for allele-specific DNA DSB repair- DNA was extracted from 4 leaves of M82 35S:Cas9 u6-26:Ps#2-sgRNA *psy1*$^{+A}$/ *psy1*$^{+A}$, *S. pimpinellifolium*^{LA1578} and 5 plants of their F_1 inbred. illumina sequencing was preformed and each pie represent a summary of 600,000-900,000 reads per plant.

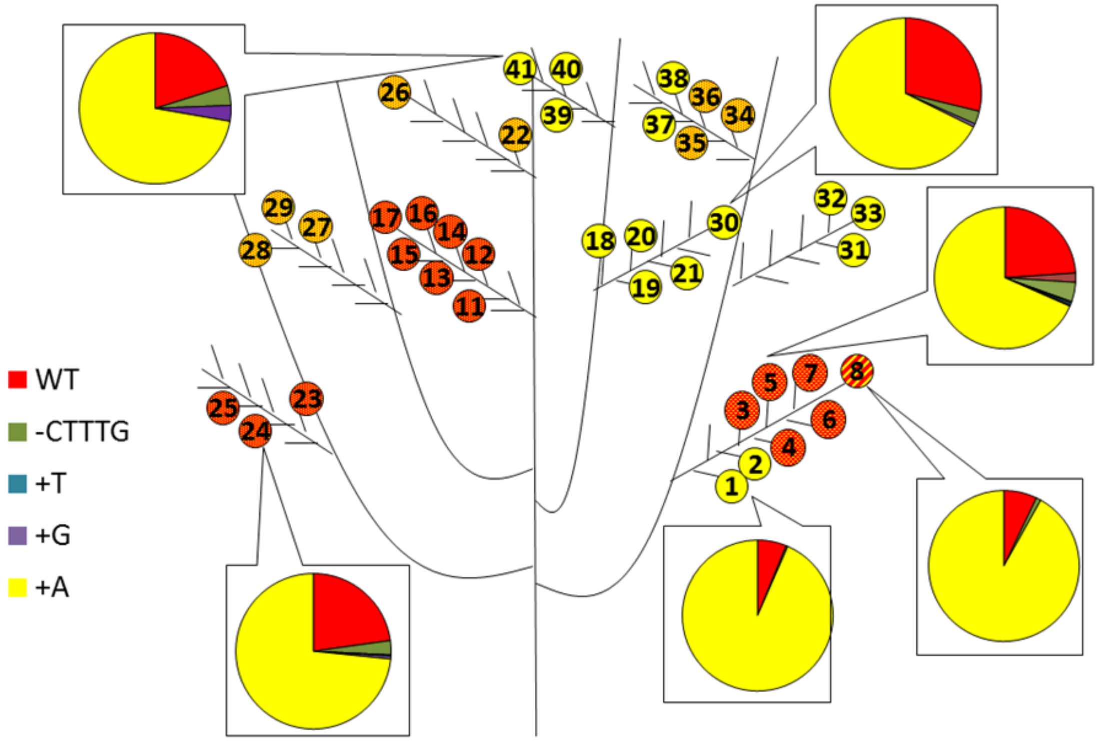

Supplementary Figure 6 - A schematic map of Fruit color phenotypes throughout development and sequencing of DNA DSB repair footprints from fruit pericarp tissues using illumina sequencing. An example is shown for plant#1, which is a F_1 plant of M82 35S:Cas9 u6-26:Ps#2-sgRNA *psy1*$^{+A}$/ *psy1*$^{+A}$ x *S. pimpinellifolium*^{LA1578} . Fruit color phenotype was variable from red through red with small or big yellow sectors to yellow. Each pie was built out of 15,000-50,000 illumina sequencing reads per fruit.

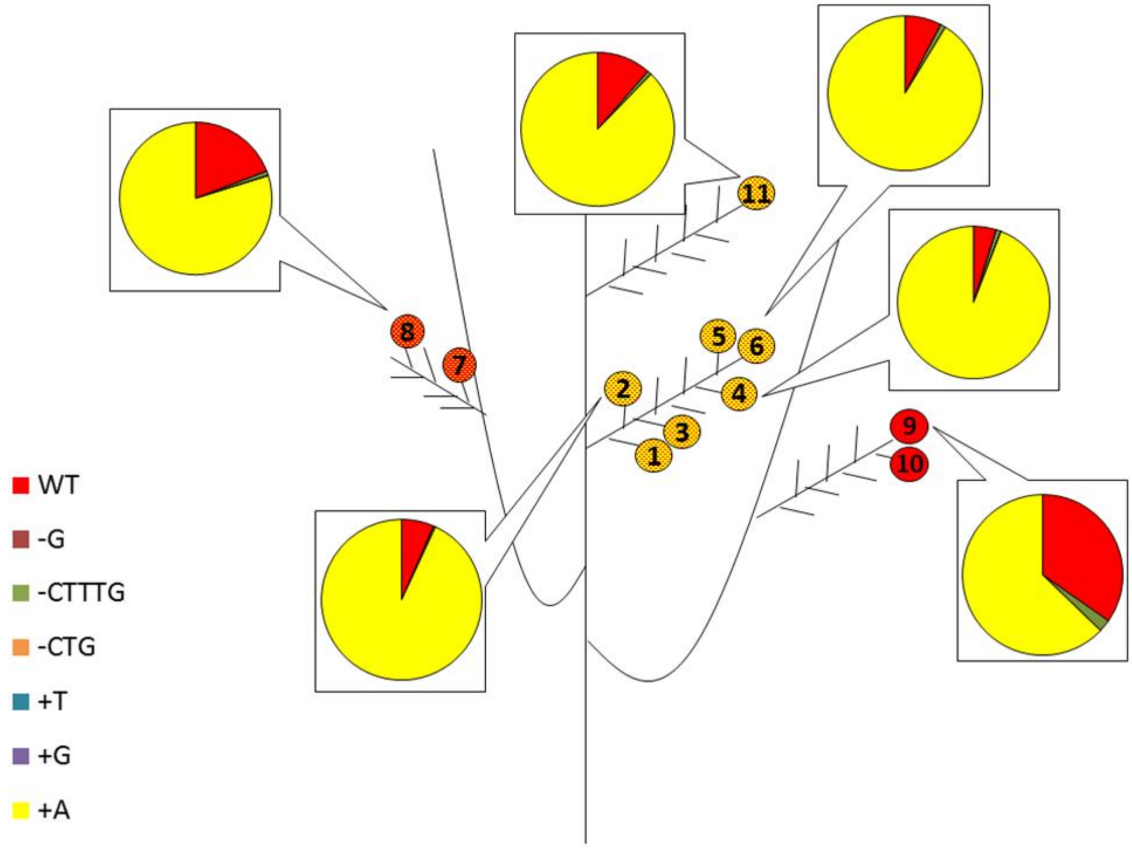

Supplementary Figure 7 - DNA DSB repair event followed by fruit phenotype and pericarp specific illumina sequencing- plant#2. All details are similar to figure S6. This plant showed high level of *psy1*$^{+A}$. The conversion products in figure 2B are the progeny of this plant.

Supplementary Table 1- Primers list:

**Primers for sgRNA targets:**	
**Ps#1 sgRNA F**	attgGAATGTCTGTTGCCTTGTTA
**Ps#1 sgRNA R**	aaacTAACAAGGCAACAGACATT
**Ps#2 sgRNA F**	attgGAGCGTATATAATGCTGCTT
**Ps#2 sgRNA R**	aaacAAGCAGCATTATATACGCT
**Primers for inverse PCR HR detection (allele specific primers):**	
**a 3756 bic hr f**	tcagCTATGCTAATGACTCCCGAG
**a bic hr r**	agtcCATTCTCTATTCCGCATAGTGA
**a 3756 r**	tgacAACCGACCTAAATCGATCCG
**b bic hr r**	actgCATTCTCTATTCCGCATAGTGA
**b 3756 r**	gactactgAACCGACCTAAATCGATCCG
**Primers for synthetic crossover - control plasmids :**	
**pupd_y1_f**	gcgccgtctcgctcgtactCGAACGAGGGTCATC
**pupd_y1_r**	gcgccgtctcgctcgagcgCCATAATTGGAACACTCATCAA
**pupd_ps_f**	gcgccgtctcgctcgggagCAACCTTATTTTGTACTT
**pupd_ps_r**	gcgccgtctcgctcgagtaCAACATATCAAAATAGGTAT
**Primers for NHEJ High throughput sequencing**	
**psy1 t1 htp f**	GGTTTGCCTGTCTGTGGTCT
**psy1 t1 htp r1**	agtcCCATGAAACTTGTCCCATTTG
**psy1 t1 htp r2**	tcagCCATGAAACTTGTCCCATTTG
**psy1 t1 htp r3**	actgCCATGAAACTTGTCCCATTTG
**psy1 t1 htp r4**	tgacCCATGAAACTTGTCCCATTTG
**psy1 t1 htp r5**	gactCCATGAAACTTGTCCCATTTG
**psy1 t1 htp r6**	ctgaCCATGAAACTTGTCCCATTTG
**nhej_psy1_t2_r**	GCCTAAATACGGCACTTCCA
**a_nhej_psy1_t2_f**	agtcGTATCGCCCCTGAATCAAAG
**b_nhej_psy1_t2_f**	tcagGTATCGCCCCTGAATCAAAG
**c_nhej_psy1_t2_f**	tgacGTATCGCCCCTGAATCAAAG
**d_nhej_psy1_t2_f**	actgGTATCGCCCCTGAATCAAAG
**e_nhej_psy1_t2_f**	gactGTATCGCCCCTGAATCAAAG
**f_nhej_psy1_t2_f**	ctgaGTATCGCCCCTGAATCAAAG
**nnn_a_nhej_psy1_t2_f**	nnnnnnagtcGTATCGCCCCTGAATCAAAG
**nnn_b_nhej_psy1_t2_f**	nnnnnntcagGTATCGCCCCTGAATCAAAG
**nnn_nhej_psy1_t2_r**	nnnnnngcctAAATACGGCACTTCCA
**Primers for Sanger sequencing of psy1 allele (SNPs detection)**	
**PSY1_1_F**	tttgcagaagtcaagaaacagg
**PSY1_t4_ident_R**	gatgtcatcgtccgttctcc
**PSY1 psnps F**	acggtatcttcccaccttca
**PSY1 psnps R2**	atagtgttaattgtgtaggctcctt

**PSY1 psnps F2**	cgacgaggagtaaggtttgc
**PSY1 psnps R**	tcagtccatttcgttttcgt
**psy1_t123_f**	atgttgcagccattcagaga
**psy1_t123_r**	tgatcatggctcgtcactgt
**psy1 term f**	acaagtaccctgggttggag
**psy1_term_r2**	gcagtttttgtaggaggcaca
**psy1_term_f2**	tgtgcctcctacaaaaactgc
**psy1 term r**	tggattgaatcgaatttggataa
**pimpixm82_co_f**	ctttgcacttggttactcaga
**pb_psy1_r**	agcctacggcccaaactatt
**14036_f**	tgctaatggggcaggaaata
**14036_r**	tcaagtaacgtaaaacacgttgaaa
**5kb_up_t2_F**	ttcatttgacgagcgatctg
**5kb_up_t2_R**	ttggctgctttgaccttacc
**40kb_down_t2_f**	cattatcctaagagtgcagtcagc
**40kb_down_t2_r**	tggtttctcgattacctctttca
**20kb_down_t2_f**	tgacaccaatccatccaatc
**20kb_down_t2_r**	ctgctacctgcactggctct
**20kb_up_t2_f**	tacgtccccgaagaaatcac
**20kb_up_t2_r**	cccttaggctccgaagttgt
**40kb_up_t2_f**	cacataagaggacacgtttattca
**40kb_up_t2_r**	gccacggagaaaatagttga
**Primers for NHEJ germinal events estimation (allele specific bends*):**	
**JF_F**	tgcaaagtgctacgtgtcct
**PSY1_1_R**	aatgtgaacagcaacgcaaa

* This set of primers give 2 bends. We extracted each of them from gel and sequence.

Supplementary Note 1 - Plasmids list:

Plasmids for Tomato fruit color assay:

35S:Cas9 plasmid:

CaMV35S, atCas9, OCS terminator

>
GGGGACAAGTTTGTACAAAAAAGCAGGCTATGGATCCCCGGGATCATCTACTTCTGAAGACTCAGACTCAGACTAAGCAGGTGAC
GAACGTCACCAATCCCAATTCGATCTACATCGATAAGAAGTACTCTATCGGACTCGATATCGGAACTAACTCTGTGGGATGGGCTG
TGATCACCGATGAGTACAAGGTGCCATCTAAGAAGTTCAAGGTTCTCGGAAACACCGATAGGCACTCTATCAAGAAAAACCTTAT
CGGTGCTCTCCTCTTCGATTCTGGTGAAACTGCTGAGGCTACCAGACTCAAGAGAACCGCTAGAAGAAGGTACACCAGAAGAAAG
AACAGGATCTGCTACCTCCAAGAGATCTTCTCTAACGAGATGGCTAAAGTGGATGATTCATTCTTCCACAGGCTCGAAGAGTCATT
CCTCGTGGAAGAAGATAAGAAGCACGAGAGGCACCCTATCTTCGGAAACATCGTTGATGAGGTGGCATACCACGAGAAGTACCC
TACTATCTACCACCTCAGAAAGAAGCTCGTTGATTCTACTGATAAGGCTGATCTCAGGCTCATCTACCTCGCTCTCGCTCACATGAT
CAAGTTCAGAGGACACTTCCTCATCGAGGGTGATCTCAACCCTGATAACTCTGATGTGGATAAGTTGTTCATCCAGCTCGTGCAGA
CCTACAACCAGCTTTTCGAAGAGAACCCTATCAACGCTTCAGGTGTGGATGCTAAGGCTATCCTCTCTGCTAGGCTCTCTAAGTCAA
GAAGGCTTGAGAACCTCATTGCTCAGCTCCCTGGTGAGAAGAAGAACGGACTTTTCGGAAACTTGATCGCTCTCTCTCTCGGACTC
ACCCCTAACTTCAAGTCTAACTTCGATCTCGCTGAGGATGCAAAGCTCCAGCTCTCAAAGGATACCTACGATGATGATCTCGATAA
CCTCCTCGCTCAGATCGGAGATCAGTACGCTGATTTGTTCCTCGCTGCTAAGAACCTCTCTGATGCTATCCTCCTCAGTGATATCCTC
AGAGTGAACACCGAGATCACCAAGGCTCCACTCTCAGCTTCTATGATCAAGAGATACGATGAGCACCACCAGGATCTCACACTTCT
CAAGGCTCTTGTTAGACAGCAGCTCCCAGAGAAGTACAAAGAGATTTTCTTCGATCAGTCTAAGAACGGATACGCTGGTTACATC
GATGGTGGTGCATCTCAAGAAGAGTTCTACAAGTTCATCAAGCCTATCCTCGAGAAGATGGATGGAACCGAGGAACTCCTCGTGA
AGCTCAATAGAGAGGATCTTCTCAGAAAGCAGAGGACCTTCGATAACGGATCTATCCCTCATCAGATCCACCTCGGAGAGTTGCA
CGCTATCCTTAGAAGGCAAGAGGATTTCTACCCATTCCTCAAGGATAACAGGGAAAAGATTGAGAAGATTCTCACCTTCAGAATCC
CTTACTACGTGGGACCTCTCGCTAGAGGAAACTCAAGATTCGCTTGGATGACCAGAAAGTCTGAGGAAACCATCACCCCTTGGAA
CTTCGAAGAGGTGGTGGATAAGGGTGCTAGTGCTCAGTCTTTCATCGAGAGGATGACCAACTTCGATAAGAACCTTCCAAACGAG
AAGGTGCTCCCTAAGCACTCTTTGCTCTACGAGTACTTCACCGTGTACAACGAGTTGACCAAGGTTAAGTACGTGACCGAGGGAAT
GAGGAAGCCTGCTTTTTTGTCAGGTGAGCAAAAGAAGGCTATCGTTGATCTCTTGTTCAAGACCAACAGAAAGGTGACCGTGAAG
CAGCTCAAAGAGGATTACTTCAAGAAAATCGAGTGCTTCGATTCAGTTGAGATTTCTGGTGTTGAGGATAGGTTCAACGCATCTCT
CGGAACCTACCACGATCTCCTCAAGATCATTAAGGATAAGGATTTCTTGGATAACGAGGAAAACGAGGATATCTTGGAGGATATC
GTTCTTACCCTCACCCTCTTTGAAGATAGAGAGATGATTGAAGAAAGGCTCAAGACCTACGCTCATCTCTTCGATGATAAGGTGAT
GAAGCAGTTGAAGAGAAGAAGATACACTGGTTGGGGAAGGCTCTCAAGAAAGCTCATTAACGGAATCAGGGATAAGCAGTCTG
GAAAGACAATCCTTGATTTCCTCAAGTCTGATGGATTCGCTAACAGAAACTTCATGCAGCTCATCCACGATGATTCTCTCACCTTTA
AAGAGGATATCCAGAAGGCTCAGGTTTCAGGACAGGGTGATAGTCTCCATGAGCATATCGCTAACCTCGCTGGATCTCCTGCAAT
CAAGAAGGGAATCCTCCAGACTGTGAAGGTTGTGGATGAGTTGGTGAAGGTGATGGGAAGGCATAAGCCTGAGAACATCGTGA
TCGAAATGGCTAGAGAGAACCAGACCACTCAGAAGGGACAGAAGAACTCTAGGGAAAGGATGAAGAGGATCGAGGAAGGTATC
AAAGAGCTTGGATCTCAGATCCTCAAAGAGCACCCTGTTGAGAACACTCAGCTCCAGAATGAGAAGCTCTACCTCTACTACCTCCA
GAACGGAAGGGATATGTATGTGGATCAAGAGTTGGATATCAACAGGCTCTCTGATTACGATGTTGATCATATCGTGCCACAGTCA
TTCTTGAAGGATGATTCTATCGATAACAAGGTGCTCACCAGGTCTGATAAGAACAGGGGTAAGAGTGATAACGTGCCAAGTGAA
GAGGTTGTGAAGAAAATGAAGAACTATTGGAGGCAGCTCCTCAACGCTAAGCTCATCACTCAGAGAAAGTTCGATAACTTGACTA
AGGCTGAGAGGGGAGGACTCTCTGAATTGGATAAGGCAGGATTCATCAAGAGGCAGCTTGTGGAAACCAGGCAGATCACTAAG
CACGTTGCACAGATCCTCGATTCTAGGATGAACACCAAGTACGATGAGAACGATAAGTTGATCAGGGAAGTGAAGGTTATCACCC
TCAAGTCAAAGCTCGTGTCTGATTTCAGAAAGGATTTCCAATTCTACAAGGTGAGGGAAATCAACAACTACCACCACGCTCACGAT
GCTTACCTTAACGCTGTTGTTGGAACCGCTCTCATCAAGAAGTATCCTAAGCTCGAGTCAGAGTTCGTGTACGGTGATTACAAGGT
GTACGATGTGAGGAAGATGATCGCTAAGTCTGAGCAAGAGATCGGAAAGGCTACCGCTAAGTATTTCTTCTACTCTAACATCATG
AATTTCTTCAAGACCGAGATTACCCTCGCTAACGGTGAGATCAGAAAGAGGCCACTCATCGAGACAAACGGTGAAACAGGTGAG
ATCGTGTGGGATAAGGGAAGGGATTTCGCTACCGTTAGAAAGGTGCTCTCTATGCCACAGGTGAACATCGTTAAGAAAACCGAG
GTGCAGACCGGTGGATTCTCTAAAGAGTCTATCCTCCCTAAGAGGAACTCTGATAAGCTCATTGCTAGGAAGAAGGATTGGGACC

CTAAGAAATACGGTGGTTTCGATTCTCCTACCGTGGCTTACTCTGTTCTCGTTGTGGCTAAGGTTGAGAAGGGAAAGAGTAAGAA
GCTCAAGTCTGTTAAGGAACTTCTCGGAATCACTATCATGGAAAGGTCATCTTTCGAGAAGAACCCAATCGATTTCCTCGAGGCTA
AGGGATACAAAGAGGTTAAGAAGGATCTCATCATCAAGCTCCCAAAGTACTCACTCTTCGAACTCGAGAACGGTAGAAAGAGGAT
GCTCGCTTCTGCTGGTGAGCTTCAAAAGGGAAACGAGCTTGCTCTCCCATCTAAGTACGTTAACTTTCTTTACCTCGCTTCTCACTA
CGAGAAGTTGAAGGGATCTCCAGAAGATAACGAGCAGAAGCAACTTTTCGTTGAGCAGCACAAGCACTACTTGGATGAGATCAT
CGAGCAGATCTCTGAGTTCTCTAAAAGGGTGATCCTCGCTGATGCAAACCTCGATAAGGTGTTGTCTGCTTACAACAAGCACAGA
GATAAGCCTATCAGGGAACAGGCAGAGAACATCATCCATCTCTTCACCCTTACCAACCTCGGTGCTCCTGCTGCTTTCAAGTACTTC
GATACAACCATCGATAGGAAGAGATACACCTCTACCAAAGAAGTGCTCGATGCTACCCTCATCCATCAGTCTATCACTGGACTCTA
CGAGACTAGGATCGATCTCTCACAGCTCGGTGGTGATTCAAGGGCTGATCCTAAGAAGAAGAGGAAGGTTTGAACCCAGCTTTCT
TGTACAAAGTGGGGGTTCGAAATCGATAAGCTTGGATCCTCTAGAGTCCTGCTTTAATGAGATATGCGAGACGCCTATGATCGCA
TGATATTTGCTTTCAATTCTGTTGTGCACGTTGTAAAAAACCTGAGCATGTGTAGCTCAGATCCTTACCGCCGGTTTCGGTTCATTCT
AATGAATATATCACCCGTTACTATCGTATTTTTATGAATAATATTCTCCGTTCAATTTACTGATTGTACCCTACTACTTATATGTACAA
TATTAAAATGAAAACAATATATTGTGCTGAATAGGTTTATAGCGACATCTATGATAGAGCGCCACAATAACAAACAATTGCGTTTT
ATTATTACAAATCCAATTTTAAAAAAAGCGGCAGAACCGGTCAAACCTAAAAGACTGATTACATAAATCTTATTCAAATTTCAAAA
GGCCCCAGGGGCTAGTATCTACGACACACCGAGCGGCGAACTAATAACGTTCACTGAAGGGAACTCCGGTTCCCCGCCGGCGCG
CATGGGTGAGATTCCTTGAAGTTGAGTATTGGCCGTCCGCTCTACCGAAAGTTACGGGCACCATTCAACCCGGTCCAGCACGGCG
GCCGGGTAACCGACTTGCTGCCCCGAGAATTATGCAGCATTTTTTTGGTGTATGTGGGCCCCAAATGAAGTGCAGGTCAAACCTT
GACAGTGACGACAAATCGTTGGGCGGGTCCAGGGCGAATTTTGCGACAACATGTCGAGGCTCAGCAGGACCTGCAGGCATGCAA
GCTAGCTTACTAGTGATGCATATTCTATAGTGTCACCTAAATCTGCGGCCGCACTAGTGATATCCCGCGGCCATGGCGGCCGGGA
GCATGCGACGTCGGGCCCAATTCGCCCTATAGTGAGTCGTATTACAATTCACTGGCCGTCGTTTTACAACGTCGTGACTGGGAAAA
CCCTGGCGTTACCCAACTTAATCGCCTTGCAGCACATCCCCCTTTCGCCAGCTGGCGTAATAGCGAAGAGGCCCGCACCGATCGCC
CTTCCCAACAGTTGCGCAGCCTGAATGGCGAATGGAAATTGTAAACGTTAATGGGTTTCTGGAGTTTAATGAGCTAAGCACATAC
GTCAGAAACCATTATTGCGCGTTCAAAAGTCGCCTAAGGTCACTATCAGCTAGCAAATATTTCTTGTCAAAAATGCTCCACTGACGT
TCCATAAATTCCCCTCGGTATCCAATTAGAGTCTCATATTCACTCTCAATCCAAATAATCTGCAATGGCAATTACCTTATCCGCAACT
TCTTTACCTATTTCCGCCCGGATCCGGGCAGGTTCTCCGGCCGCTTGGGTGGAGAGGCTATTCGGCTATGACTGGGCACAACAGA
CAATCGGCTGCTCTGATGCCGCCGTGTTCCGGCTGTCAGCGCAGGGGCGCCCGGTTCTTTTTGTCAAGACCGACCTGTCCGGTGCC
CTGAATGAACTGCAGGACGAGGCAGCGCGGCTATCGTGGCTGGCCACGACGGGCGTTCCTTGCGCAGCTGTGCTCGACGTTGTC
ACTGAAGCGGGAAGGGACTGGCTGCTATTGGGCGAAGTGCCGGGGCAGGATCTCCTGTCATCTCACCTTGCTCCTGCCGAGAAA
GTATCCATCATGGCTGATGCAATGCGGCGGCTGCATACGCTTGATCCGGCTACCTGCCCATTCGACCACCAAGCGAAACATCGCAT
CGAGCGAGCACGTACTCGGATGGAAGCCGGTCTTGTCGATCAGGATGATCTGGACGAAGAGCATCAGGGGCTCGCGCCAGCCGA
ACTGTTCGCCAGGCTCAAGGCGCGCATGCCCGACGGCGAGGATCTCGTCGTGACCCATGGCGATGCCTGCTTGCCGAATATCATG
GTGGAAAATGGCCGCTTTTCTGGATTCATCGACTGTGGCCGGCTGGGTGTGGCGGACCGCTATCAGGACATAGCGTTGGCTACCC
GTGATATTGCTGAAGAGCTTGGCGGCGAATGGGCTGACCGCTTCCTCGTGCTTTACGGTATCGCCGCTCCCGATTCGCAGCGCATC
GCCTTCTATCGCCTTCTTGACGAGTTCTTCTGAGCGGGACTCTGGGGTTCGAAATGACCGACCAAGCGACGCCCAACCTGCCATCA
CGAGATTTCGATTCCACCGCCGCCTTCTATGAAAGGTTGGGCTTCGGAATCGTTTTCCGGGACGCCGGCTGGATGATCCTCCAGCG
CGGGGATCTCATGCTGGAGTTCTTCGCCCACCCCGATCCAACACTTACGTTTGCAACGTCCAAGAGCAAATAGACCACGAACGCCG
GAAGGTTGCCGCAGCGTGTGGATTGCGTCTCAATTCTCTCTTGCAGGAATGCAATGATGAATATGATACTGACTATGAAACTTTGA
GGGAATACTGCCTAGCACCGTCACCTCATAACGTGCATCATGCATGCCCTGACAACATGGAACATCGCTATTTTTCTGAAGAATTA
TGCTCGTTGGAGGATGTCGCGGCAATTGCAGCTATTGCCAACATCGAACTACCCCTCACGCATGCATTCATCAATATTATTCATGCG
GGGAAAGGCAAGATTAATCCAACTGGCAAATCATCCAGCGTGATTGGTAACTTCAGTTCCAGCGACTTGATTCGTTTTGGTGCTAC
CCACGTTTTCAATAAGGACGAGATGGTGGAGTAAAGAAGGAGTGCGTCGAAGCAGATCGTTCAAACATTTGGCAATAAAGTTTCT
TAAGATTGAATCCTGTTGCCGGTCTTGCGATGATTATCATATAATTTCTGTTGAATTACGTTAAGCATGTAATAATTAACATGTAAT
GCATGACGTTATTTATGAGATGGGTTTTTATGATTAGAGTCCCGCAATTATACATTTAATACGCGATAGAAAACAAAATATAGCGC
GCAAACTAGGATAAATTATCGCGCGCGGTGTCATCTATGTTACTAGATCGAATTAATTCAGTACATTAAAAACGTCCGCAATGTGT
TATTAAGTTGTCTAAGCGTCAATTTGTTTACACCACAATATATCCTGCCACCAGCCAGCCAACAGCTCCCCGACCGGCAGCTCGGCA
CAAAATCACCACTCGATACAGGCAGCCCATCAGTCCGGGACGGCGTCAGCGGGAGAGCCGTTGTAAGGCGGCAGACTTTGCTCA
TGTTACCGATGCTATTCGGAAGAACGGCAACTAAGCTGCCGGGTTTGAAACACGGATGATCTCGCGGAGGGTAGCATGTTGATTG
TAACGATGACAGAGCGTTGCTGCCTGTGATCAAATATCATCTCCCTCGCAGAGATCCGAATTATCAGCCTTCTTATTCATTTCTCGC
TTAACCGTGACAGGCTGTCGATCTTGAGAACTATGCCGACATAATAGGAAATCGCTGGATAAAGCCGCTGAGGAAGCTGAGTGG
CGCTATTTCTTTAGAAGTGAACGTTGACGATGTCGACGGATCTTTTCCGCTGCATAACCCTGCTTCGGGGTCATTATAGCGATTTTT
TCGGTATATCCATCCTTTTTCGCACGATATACAGGATTTTGCCAAAGGGTTCGTGTAGACTTTCCTTGGTGTATCCAACGGCGTCAG

CCGGGCAGGATAGGTGAAGTAGGCCCACCCGCGAGCGGGTGTTCCTTCTTCACTGTCCCTTATTCGCACCTGGCGGTGCTCAACG
GGAATCCTGCTCTGCGAGGCTGGCCGGCTACCGCCGGCGTAACAGATGAGGGCAAGCGGATGGCTGATGAAACCAAGCCAACCA
GGGGTGATGCTGCCAACTTACTGATTTAGTGTATGATGGTGTTTTTGAGGTGCTCCAGTGGCTTCTGTTTCTATCAGCTGTCCCTCC
TGTTCAGCTACTGACGGGGTGGTGCGTAACGGCAAAAGCACCGCCGGACATCAGCGCTATCTCTGCTCTCACTGCCGTAAAACAT
GGCAACTGCAGTTCACTTACACCGCTTCTCAACCCGGTACGCACCAGAAAATCATTGATATGGCCATGAATGGCGTTGGATGCCG
GGCAACAGCCCGCATTATGGGCGTTGGCCTCAACACGATTTTACGTCACTTAAAAAACTCAGGCCGCAGTCGGTAACCTCGCGCAT
ACAGCCGGGCAGTGACGTCATCGTCTGCGCGGAAATGGACGAACAGTGGGGCTATGTCGGGGCTAAATCGCGCCAGCGCTGGCT
GTTTTACGCGTATGACAGTCTCCGGAAGACGGTTGTTGCGCACGTATTCGGTGAACGCACTATGGCGACGCTGGGGCGTCTTATG
AGCCTGCTGTCACCCTTTGACGTGGTGATATGGATGACGGATGGCTGGCCGCTGTATGAATCCCGCCTGAAGGGAAAGCTGCACG
TAATCAGCAAGCGATATACGCAGCGAATTGAGCGGCATAACCTGAATCTGAGGCAGCACCTGGCACGGCTGGGACGGAAGTCGC
TGTCGTTCTCAAAATCGGTGGAGCTGCATGACAAAGTCATCGGGCATTATCTGAACATAAAACACTATCAATAAGTTGGAGTCATT
ACCCAACCAGGAAGGGCAGCCCACCTATCAAGGTGTACTGCCTTCCAGACGAACGAAGAGCGATTGAGGAAAAGGCGGCGGCG
GCCGGCATGAGCCTGTCGGCCTACCTGCTGGCCGTCGGCCAGGGCTACAAAATCACGGGCGTCGTGGACTATGAGCACGTCCGC
GAGCTGGCCCGCATCAATGGCGACCTGGGCCGCCTGGGCGGCCTGCTGAAACTCTGGCTCACCGACGACCCGCGCACGGCGCGG
TTCGGTGATGCCACGATCCTCGCCCTGCTGGCGAAGATCGAAGAGAAGCAGGACGAGCTTGGCAAGGTCATGATGGGCGTGGTC
CGCCCGAGGGCAGAGCCATGACTTTTTTAGCCGCTAAAACGGCCGGGGGGTGCGCGTGATTGCCAAGCACGTCCCCATGCGCTCC
ATCAAGAAGAGCGACTTCGCGGAGCTGGTATTCGTGCAGGGCAAGATTCGGAATACCAAGTACGAGAAGGACGGCCAGACGGT
CTACGGGACCGACTTCATTGCCGATAAGGTGGATTATCTGGACACCAAGGCACCAGGCGGGTCAAATCAGGAATAAGGGCACAT
TGCCCCGGCGTGAGTCGGGGCAATCCCGCAAGGAGGGTGAATGAATCGGACGTTTGACCGGAAGGCATACAGGCAAGAACTGA
TCGACGCGGGGTTTTCCGCCGAGGATGCCGAAACCATCGCAAGCCGCACCGTCATGCGTGCGCCCCGCGAAACCTTCCAGTCCGT
CGGCTCGATGGTCCAGCAAGCTACGGCCAAGATCGAGCGCGACAGCGTGCAACTGGCTCCCCCTGCCCTGCCCGCGCCATCGGCC
GCCGTGGAGCGTTCGCGTCGTCTCGAACAGGAGGCGGCAGGTTTGGCGAAGTCGATGACCATCGACACGCGAGGAACTATGACG
ACCAAGAAGCGAAAAACCGCCGGCGAGGACCTGGCAAAACAGGTCAGCGAGGCCAAGCAGGCCGCGTTGCTGAAACACACGAA
GCAGCAGATCAAGGAAATGCAGCTTTCCTTGTTCGATATTGCGCCGTGGCCGGACACGATGCGAGCGATGCCAAACGACACGGC
CCGCTCTGCCCTGTTCACCACGCGCAACAAGAAAATCCCGCGCGAGGCGCTGCAAAACAAGGTCATTTTCCACGTCAACAAGGAC
GTGAAGATCACCTACACCGGCGTCGAGCTGCGGGCCGACGATGACGAACTGGTGTGGCAGCAGGTGTTGGAGTACGCGAAGCG
CACCCCTATCGGCGAGCCGATCACCTTCACGTTCTACGAGCTTTGCCAGGACCTGGGCTGGTCGATCAATGGCCGGTATTACACGA
AGGCCGAGGAATGCCTGTCGCGCCTACAGGCGACGGCGATGGGCTTCACGTCCGACCGCGTTGGGCACCTGGAATCGGTGTCGC
TGCTGCACCGCTTCCGCGTCCTGGACCGTGGCAAGAAAACGTCCCGTTGCCAGGTCCTGATCGACGAGGAAATCGTCGTGCTGTT
TGCTGGCGACCACTACACGAAATTCATATGGGAGAAGTACCGCAAGCTGTCGCCGACGGCCCGACGGATGTTCGACTATTTCAGC
TCGCACCGGGAGCCGTACCCGCTCAAGCTGGAAACCTTCCGCCTCATGTGCGGATCGGATTCCACCCGCGTGAAGAAGTGGCGCG
AGCAGGTCGGCGAAGCCTGCGAAGAGTTGCGAGGCAGCGGCCTGGTGGAACACGCCTGGGTCAATGATGACCTGGTGCATTGC
AAACGCTAGGGCCTTGTGGGGTCAGTTCCGGCTGGGGGTTCAGCAGCCAGCGCTTTACTGGCATTTCAGGAACAAGCGGGCACT
GCTCGACGCACTTGCTTCGCTCAGTATCGCTCGGGACGCACGGCGCGCTCTACGAACTGCCGATAAACAGAGGATTAAAATTGAC
AATTGTGATTAAGGCTCAGATTCGACGGCTTGGAGCGGCCGACGTGCAGGATTTCCGCGAGATCCGATTGTCGGCCCTGAAGAAA
GCTCCAGAGATGTTCGGGTCCGTTTACGAGCACGAGGAGAAAAAGCCCATGGAGGCGTTCGCTGAACGGTTGCGAGATGCCGTG
GCATTCGGCGCCTACATCGACGGCGAGATCATTGGGCTGTCGGTCTTCAAACAGGAGGACGGCCCCAAGGACGCTCACAAGGCG
CATCTGTCCGGCGTTTTCGTGGAGCCCGAACAGCGAGGCCGAGGGGTCGCCGGTATGCTGCTGCGGGCGTTGCCGGCGGGTTTA
TTGCTCGTGATGATCGTCCGACAGATTCCAACGGGAATCTGGTGGATGCGCATCTTCATCCTCGGCGCACTTAATATTTCGCTATTC
TGGAGCTTGTTGTTTATTTCGGTCTACCGCCTGCCGGGCGGGGTCGCGGCGACGGTAGGCGCTGTGCAGCCGCTGATGGTCGTGT
TCATCTCTGCCGCTCTGCTAGGTAGCCCGATACGATTGATGGCGGTCCTGGGGGCTATTTGCGGAACTGCGGGCGTGGCGCTGTT
GGTGTTGACACCAAACGCAGCGCTAGATCCTGTCGGCGTCGCAGCGGGCCTGGCGGGGGCGGTTTCCATGGCGTTCGGAACCGT
GCTGACCCGCAAGTGGCAACCTCCCGTGCCTCTGCTCACCTTTACCGCCTGGCAACTGGCGGCCGGAGGACTTCTGCTCGTTCCAG
TAGCTTTAGTGTTTGATCCGCCAATCCCGATGCCTACAGGAACCAATGTTCTCGGCCTGGCGTGGCTCGGCCTGATCGGAGCGGG
TTTAACCTACTTCCTTTGGTTCCGGGGGATCTCGCGACTCGAACCTACAGTTGTTTCCTTACTGGGCTTTCTCAGCCGGGATGGCGC
TAAGAAGCTATTGCCGCCGATCTTCATATGCGGTGTGAAATACCGCACAGATGCGTAAGGAGAAAATACCGCATCAGGCGCTCTT
CCGCTTCCTCGCTCACTGACTCGCTGCGCTCGGTCGTTCGGCTGCGGCGAGCGGTATCAGCTCACTCAAAGGCGGTAATACGGTTA
TCCACAGAATCAGGGGATAACGCAGGAAAGAACATGTGAGCAAAAGGCCAGCAAAAGGCCAGGAACCGTAAAAAGGCCGCGTT
GCTGGCGTTTTTCCATAGGCTCCGCCCCCCTGACGAGCATCACAAAAATCGACGCTCAAGTCAGAGGTGGCGAAACCCGACAGGA
CTATAAAGATACCAGGCGTTTCCCCCTGGAAGCTCCCTCGTGCGCTCTCCTGTTCCGACCCTGCCGCTTACCGGATACCTGTCCGCC
TTTCTCCCTTCGGGAAGCGTGGCGCTTTCTCAATGCTCACGCTGTAGGTATCTCAGTTCGGTGTAGGTCGTTCGCTCCAAGCTGGG

CTGTGTGCACGAACCCCCCGTTCAGCCCGACCGCTGCGCCTTATCCGGTAACTATCGTCTTGAGTCCAACCCGGTAAGACACGACT
TATCGCCACTGGCAGCAGCCACTGGTAACAGGATTAGCAGAGCGAGGTATGTAGGCGGTGCTACAGAGTTCTTGAAGTGGTGGC
CTAACTACGGCTACACTAGAAGGACAGTATTTGGTATCTGCGCTCTGCTGAAGCCAGTTACCTTCGGAAAAAGAGTTGGTAGCTCT
TGATCCGGCAAACAAACCACCGCTGGTAGCGGTGGTTTTTTTGTTTGCAAGCAGCAGATTACGCGCAGAAAAAAAGGATATCAAG
AAGATCCTTTGATCTTTTCTACGGGGTCTGACGCTCAGTGGAACGAAAACTCACGTTAAGGGATTTTGGTCATGAGATTATCAAAA
AGGATCTTCACCTAGATCCTTTTAAATTAAAAATGAAGTTTTAAATCAATCTAAAGTATATATGAGTAAACTTGGTCTGACAGTTAC
CAATGCTTAATCAGTGAGGCACCTATCTCAGCGATCTGTCTATTTCGTTCATCCATAGTTGCCTGACTCCCCGTCGTGTAGATAACT
ACGATACGGGAGGGCTTACCATCTGGCCCCAGTGCTGCAATGATACCGCGAGACCCACGCTCACCGGCTCCAGATTTATCAGCAA
TAAACCAGCCAGCCGGAAGGGCCGAGCGCAGAAGTGGTCCTGCAACTTTATCCGCCTCCATCCAGTCTATTAAACAAGTGGCAGC
AACGGATTCGCAAACCTGTCACGCCTTTTGTGCCAAAAGCCGCGCCAGGTTTGCGATCCGCTGTGCCAGGCGTTAGGCGTCATAT
GAAGATTTCGGTGATCCCTGAGCAGGTGGCGGAAACATTGGATGCTGAGAACCATTTCATTGTTCGTGAAGTGTTCGATGTGCAC
CTATCCGACCAAGGCTTTGAACTATCTACCAGAAGTGTGAGCCCCTACCGGAAGGATTACATCTCGGATGATGACTCTGATGAAG
ACTCTGCTTGCTATGGCGCATTCATCGACCAAGAGCTTGTCGGGAAGATTGAACTCAACTCAACATGGAACGATCTAGCCTCTATC
GAACACATTGTTGTGTCGCACACGCACCGAGGCAAAGGAGTCGCGCACAGTCTCATCGAATTTGCGAAAAAGTGGGCACTAAGC
AGACAGCTCCTTGGCATACGATTAGAGACACAAACGAACAATGTACCTGCCTGCAATTTGTACGCAAAATGTGGCTTTACTCTCGG
CGGCATTGACCTGTTCACGTATAAAACTAGACCTCAAGTCTCGAACGAAACAGCGATGTACTGGTACTGGTTCTCGGGAGCACAG
GATGACGCCTAACAATTCATTCAAGCCGACACCGCTTCGCGGCGCGGCTTAATTCAGGAGTTAAACATCATGAGGGAAGCGGTGA
TCGCCGAAGTATCGACTCAACTATCAGAGGTAGTTGGCGTCATCGAGCGCCATCTCGAACCGACGTTGCTGGCCGTACATTTGTAC
GGCTCCGCAGTGGATGGCGGCCTGAAGCCACACAGTGATATTGATTTGCTGGTTACGGTGACCGTAAGGCTTGATGAAACAACGC
GGCGAGCTTTGATCAACGACCTTTTGGAAACTTCGGCTTCCCCTGGAGAGAGCGAGATTCTCCGCGCTGTAGAAGTCACCATTGTT
GTGCACGACGACATCATTCCGTGGCGTTATCCAGCTAAGCGCGAACTGCAATTTGGAGAATGGCAGCGCAATGACATTCTTGCAG
GTATCTTCGAGCCAGCCACGATCGACATTGATCTGGCTATCTTGCTGACAAAAGCAAGAGAACATAGCGTTGCCTTGGTAGGTCCA
GCGGCGGAGGAACTCTTTGATCCGGTTCCTGAACAGGATCTATTTGAGGCGCTAAATGAAACCTTAACGCTATGGAACTCGCCGC
CCGACTGGGCTGGCGATGAGCGAAATGTAGTGCTTACGTTGTCCCGCATTTGGTACAGCGCAGTAACCGGCAAAATCGCGCCGAA
GGATGTCGCTGCCGACTGGGCAATGGAGCGCCTGCCGGCCCAGTATCAGCCCGTCATACTTGAAGCTAGGCAGGCTTATCTTGGA
CAAGAAGATCGCTTGGCCTCGCGCGCAGATCAGTTGGAAGAATTTGTTCACTACGTGAAAGGCGAGATCACCAAGGTAGTCGGC
AAATAATGTCTAACAATTCGTTCAAGCCGACGCCGCTTCGCGGCGCGGCTTAACTCAAGCGTTAGAGAGCTGGGGAAGACTATGC
GCGATCTGTTGAAGGTGGTTCTAAGCCTCGTACTTGCGATGGCATCGGGGCAGGCACTTGCTGACCTGCCAATTGTTTTAGTGGAT
GAAGCTCGTCTTCCCTATGACTACTCCCCATCCAACTACGACATTTCTCCAAGCAACTACGACAACTCCATAAGCAATTACGACAAT
AGTCCATCAAATTACGACAACTCTGAGAGCAACTACGATAATAGTTCATCCAATTACGACAATAGTCGCAACGGAAATCGTAGGCT
TATATATAGCGCAAATGGGTCTCGCACTTTCGCCGGCTACTACGTCATTGCCAACAATGGGACAACGAACTTCTTTTCCACATCTGG
CAAAAGGATGTTCTACACCCCAAAAGGGGGGCGCGGCGTCTATGGCGGCAAAGATGGGAGCTTCTGCGGGGCATTGGTCGTCAT
AAATGGCCAATTTTCGCTTGCCCTGACAGATAACGGCCTGAAGATCATGTATCTAAGCAACTAGCCTGCTCTCTAATAAAATGTTA
GGAGCTTGGCTGCCATTTTTGGGGTGAGGCCGTTCGCGGCCGAGGGGCGCAGCCCCTGGGGGGATGGGAGGCCCGCGTTAGCG
GGCCGGGAGGGTTCGAGAAGGGGGGGCACCCCCCTTCGGCGTGCGCGGTCACGCGCCAGGGCGCAGCCCTGGTTAAAAACAAG
GTTTATAAATATTGGTTTAAAAGCAGGTTAAAAGACAGGTTAGCGGTGGCCGAAAAACGGGCGGAAACCCTTGCAAATGCTGGA
TTTTCTGCCTGTGGACAGCCCCTCAAATGTCAATAGGTGCGCCCCTCATCTGTCAGCACTCTGCCCCTCAAGTGTCAAGGATCGCGC
CCCTCATCTGTCAGTAGTCGCGCCCCTCAAGTGTCAATACCGCAGGGCACTTATCCCCAGGCTTGTCCACATCATCTGTGGGAAACT
CGCGTAAAATCAGGCGTTTTCGCCGATTTGCGAGGCTGGCCAGCTCCACGTCGCCGGCCGAAATCGAGCCTGCCCCTCATCTGTCA
ACGCCGCGCCGGGTGAGTCGGCCCCTCAAGTGTCAACGTCCGCCCCTCATCTGTCAGTGAGGGCCAAGTTTTCCGCGAGGTATCC
ACAACGCCGGCGGCCGGCCGCGGTGTCTCGCACACGGCTTCGACGGCGTTTCTGGCGCGTTTGCAGGGCCATAGACGGCCGCCA
GCCCAGCGGCGAGGGCAACCAGCCCGGTGAGCGTCGGAAAGGGTCGACATCTTGCTGCGTTCGGATATTTTCGTGGAGTTCCCG
CCACAGACCCGGATTGAAGGCGAGATCCAGCAACTCGCGCCAGATCATCCTGTGACGGAACTTTGGCGCGTGATGACTGGCCAG
GACGTCGGCCGAAAGAGCGACAAGCAGATCACGATTTTCGACAGCGTCGGATTTGCGATCGAGGATTTTTCGGCGCTGCGCTACG
TCCGCGACCGCGTTGAGGGATCAAGCCACAGCAGCCCACTCGACCTTCTAGCCGACCCAGACGAGCCAAGGGATCTTTTTGGAAT
GCTGCTCCGTCGTCAGGCTTTCCGACGTTTGGGTGGTTGAACAGAAGTCATTATCGTACGGAATGCCAGCACTCCCGAGGGGAAC
CCTGTGGTTGGCATGCACATACAAATGGACGAACGGATAAACCTTTTCACGCCCTTTTAAATATCCGTTATTCTAATAAACGCTCTT
TTCTCTTAGGTTTACCCGCCAATATATCCTGTCAAACACTGATAGTTTAAACTGAAGGCGGGAAACGACAATCTGATCATGAGCGG
AGAATTAAGGGAGTCACGTTATGACCCCCGCCGATGACGCGGGACAAGCCGTTTTACGTTTGGAACTGACAGAACCGCAACGATT
GAAGGAGCCACTCAGCCCCAATACGCAAACCGCCTCTCCCCGCGCGTTGGCCGATTCATTAATGCAGCTGGCACGACAGGTTTCCC
GACTGGAAAGCGGGCAGTGAGCGCAACGCAATTAATGTGAGTTAGCTCACTCATTAGGCACCCCAGGCTTTACACTTTATGCTTCC

GGCTCGTATGTTGTGTGGAATTGTGAGCGGATAACAATTTCACACAGGAAACAGCTATGA CCATGATTACGCCAAGCTATTTAGG
TGACACTATAGAATACTCAAGCTATGCATCCAACGCGTTGGGAGCTCTCCCATATCGACCTGCAGGCGGCCGCTCGACGAATTAAT
TCCAATCCCACAAAAATCTGAGCTTAACAGCACAGTTGCTCCTCTCAGAGCAGAATCGGGTATTCAACACCCTCATATCAACTACTA
CGTTGTGTATAACGGTCCACATGCCGGTATATACGATGACTGGGGTTGTACAAAGGCG GCAACAAACGGCGTTCCCGGAGTTGCA
CACAAGAAATTTGCCACTATTACAGAGGCAAGAGCAGCAGCTGACGCGTACACAACAAGTCAGCAAACAGACAGGTTGAACTTCA
TCCCCAAAGGAGAAGCTCAACTCAAGCCCAAGAGCTTTGCTAAGGCCCTAACAAGCCCACCAAAGCAAAAAGCCCACTGGCTCAC
GCTAGGAACCAAAAGGCCCAGCAGTGATCCAGCCCCAAAAGAGATCTCCTTTGCCCCGG AGATTACAATGGACGATTTCCTCTATC
TTTACGATCTAGGAAGGAAGTTCGAAGGTGAAGGTGACGACACTATGTTCACCACTGATAATGAGAAGGTTAGCCTCTTCAATTTC
AGAAAGAATGCTGACCCACAGATGGTTAGAGAGGCCTACGCAGCAGGTCTCATCAAGACGATCTACCCGAGTAACAATCTCCAG
GAGATCAAATACCTTCCCAAGAAGGTTAAAGATGCAGTCAAAAGATTCAGGACTAATTG CATCAAGAACACAGAGAAAGACATAT
TTCTCAAGATCAGAAGTACTATTCCAGTATGGACGATTCAAGGCTTGCTTCATAAACCAAGGCAAGTAATAGAGATTGGAGTCTCT
AAAAAGGTAGTTCCTACTGAATCTAAGGCCATGCATGGAGTCTAAGATTCAAATCGAGGATCTAACAGAACTCGCCGTGAAGACT
GGCGAACAGTTCATACAGAGTCTTTTACGACTCAATGACAAGAAGAAAATCTTCGTCAA CATGGTGGAGCACGACACTCTGGTCT
ACTCCAAAAATGTCAAAGATACAGTCTCAGAAGACCAAAGGGCTATTGAGACTTTTCAACAAAGGATAATTTCGGGAAACCTCCTC
GGATTCCATTGCCCAGCTATCTGTCACTTCATCGAAAGGACAGTAGAAAAGGAAGGTGGCTCCTACAAATGCCATCATTGCGATA
AAGGAAAGGCTATCATTCAAGATCTCTCTGCCGACAGTGGTCCCAAAGATGGACCCCCA CCCACGAGGAGCATCGTGGAAAAAG
AAGACGTTCCAACCACGTCTTCAAAGCAAGTGGATTGATGTGACATCTCCACTGACGTAAGGGATGACGCACAATCCCACTATCCT
TCGCAAGACCCTTCCTCTATATAAGGAAGTTCATTTCATTTGGAGAGGACACGCTCGAGGAATTCGGTACCCC

U6-26*:Ps#1* gRNA plasmid

U6-26 promoter, *Ps#1* gRNA

>
ACCTAGGGAGCTCTCCCATATCGACCTGCAGGCGGCCGCACTAGTGATATCCCGCGGCCATGGCGGCCGGGAGCATGCGACGTC
GGGCCCAATTCGCCCTATAGTGAGTCGTATTACAATTCACTGGCCGTCGTTTTACAACGTCGTGACTGGGAAAACCCTGGCGTTAC
CCAACTTAATCGCCTTGCAGCACATCCCCCTTTCGCCAGCTGGCGTAATAGCGAAGAGGCCCGCACCGATCGCCCTTCCCAACAGT
TGCGCAGCCTGAATGGCGAATGGAAATTGTAAACGTTAATGGGTTTCTGGAGTTTAATGAGCTAAGCACATACGTCAGAAACCAT
TATTGCGCGTTCAAAAGTCGCCTAAGGTCACTATCAGCTAGCAAATATTTCTTGTCAAAAATGCTCCACTGACGTTCCATAAATTCC
CCTCGGTATCCAATTAGAGTCTCATATTCACTCTCAATCCAAATAATCTGCAATGGCAATTACCTTATCCGCAACTTCTTTACCTATTT
CCGCCCGGATCCGGGCAGGTTCTCCGGCCGCTTGGGTGGAGAGGCTATTCGGCTATGACTGGGCACAACAGACAATCGGCTGCT
CTGATGCCGCCGTGTTCCGGCTGTCAGCGCAGGGGCGCCCGGTTCTTTTTGTCAAGACCGACCTGTCCGGTGCCCTGAATGAACT
GCAGGACGAGGCAGCGCGGCTATCGTGGCTGGCCACGACGGGCGTTCCTTGCGCAGCTGTGCTCGACGTTGTCACTGAAGCGGG
AAGGGACTGGCTGCTATTGGGCGAAGTGCCGGGGCAGGATCTCCTGTCATCTCACCTTGCTCCTGCCGAGAAAGTATCCATCATG
GCTGATGCAATGCGGCGGCTGCATACGCTTGATCCGGCTACCTGCCCATTCGACCACCAAGCGAAACATCGCATCGAGCGAGCAC
GTACTCGGATGGAAGCCGGTCTTGTCGATCAGGATGATCTGGACGAAGAGCATCAGGGGCTCGCGCCAGCCGAACTGTTCGCCA
GGCTCAAGGCGCGCATGCCCGACGGCGAGGATCTCGTCGTGACCCATGGCGATGCCTGCTTGCCGAATATCATGGTGGAAAATG
GCCGCTTTTCTGGATTCATCGACTGTGGCCGGCTGGGTGTGGCGGACCGCTATCAGGACATAGCGTTGGCTACCCGTGATATTGC
TGAAGAGCTTGGCGGCGAATGGGCTGACCGCTTCCTCGTGCTTTACGGTATCGCCGCTCCCGATTCGCAGCGCATCGCCTTCTATC
GCCTTCTTGACGAGTTCTTCTGAGCGGGACTCTGGGGTTCGAAATGACCGACCAAGCGACGCCCAACCTGCCATCACGAGATTTC
GATTCCACCGCCGCCTTCTATGAAAGGTTGGGCTTCGGAATCGTTTTCCGGGACGCCGGCTGGATGATCCTCCAGCGCGGGGATC
TCATGCTGGAGTTCTTCGCCCACCCCGATCCAACACTTACGTTTGCAACGTCCAAGAGCAAATAGACCACGAACGCCGGAAGGTTG
CCGCAGCGTGTGGATTGCGTCTCAATTCTCTCTTGCAGGAATGCAATGATGAATATGATACTGACTATGAAACTTTGAGGGAATAC
TGCCTAGCACCGTCACCTCATAACGTGCATCATGCATGCCCTGACAACATGGAACATCGCTATTTTTCTGAAGAATTATGCTCGTTG
GAGGATGTCGCGGCAATTGCAGCTATTGCCAACATCGAACTACCCCTCACGCATGCATTCATCAATATTATTCATGCGGGGAAAGG
CAAGATTAATCCAACTGGCAAATCATCCAGCGTGATTGGTAACTTCAGTTCCAGCGACTTGATTCGTTTTGGTGCTACCCACGTTTT
CAATAAGGACGAGATGGTGGAGTAAAGAAGGAGTGCGTCGAAGCAGATCGTTCAAACATTTGGCAATAAAGTTTCTTAAGATTG
AATCCTGTTGCCGGTCTTGCGATGATTATCATATAATTTCTGTTGAATTACGTTAAGCATGTAATAATTAACATGTAATGCATGACG
TTATTTATGAGATGGGTTTTTATGATTAGAGTCCCGCAATTATACATTTAATACGCGATAGAAAACAAAATATAGCGCGCAAACTA
GGATAAATTATCGCGCGCGGTGTCATCTATGTTACTAGATCGAATTAATTCAGTACATTAAAAACGTCCGCAATGTGTTATTAAGTT
GTCTAAGCGTCAATTTGTTTACACCACAATATATCCTGCCACCAGCCAGCCAACAGCTCCCCGACCGGCAGCTCGGCACAAAATCA

CCACTCGATACAGGCAGCCCATCAGTCCGGGACGGCGTCAGCGGGAGAGCCGTTGTAAGGCGGCAGACTTTGCTCATGTTACCG
ATGCTATTCGGAAGAACGGCAACTAAGCTGCCGGGTTTGAAACACGGATGATCTCGCGGAGGGTAGCATGTTGATTGTAACGAT
GACAGAGCGTTGCTGCCTGTGATCAAATATCATCTCCCTCGCAGAGATCCGAATTATCAGCCTTCTTATTCATTTCTCGCTTAACCG
TGACAGGCTGTCGATCTTGAGAACTATGCCGACATAATAGGAAATCGCTGGATAAAGCCGCTGAGGAAGCTGAGTGGCGCTATTT
CTTTAGAAGTGAACGTTGACGATGTCGACGGATCTTTTCCGCTGCATAACCCTGCTTCGGGGTCATTATAGCGATTTTTTCGGTATA
TCCATCCTTTTTCGCACGATATACAGGATTTTGCCAAAGGGTTCGTGTAGACTTTCCTTGGTGTATCCAACGGCGTCAGCCGGGCA
GGATAGGTGAAGTAGGCCCACCCGCGAGCGGGTGTTCCTTCTTCACTGTCCCTTATTCGCACCTGGCGGTGCTCAACGGGAATCCT
GCTCTGCGAGGCTGGCCGGCTACCGCCGGCGTAACAGATGAGGGCAAGCGGATGGCTGATGAAACCAAGCCAACCAGGGGTGA
TGCTGCCAACTTACTGATTTAGTGTATGATGGTGTTTTTGAGGTGCTCCAGTGGCTTCTGTTTCTATCAGCTGTCCCTCCTGTTCAGC
TACTGACGGGGTGGTGCGTAACGGCAAAAGCACCGCCGGACATCAGCGCTATCTCTGCTCTCACTGCCGTAAAACATGGCAACTG
CAGTTCACTTACACCGCTTCTCAACCCGGTACGCACCAGAAAATCATTGATATGGCCATGAATGGCGTTGGATGCCGGGCAACAG
CCCGCATTATGGGCGTTGGCCTCAACACGATTTTACGTCACTTAAAAAACTCAGGCCGCAGTCGGTAACCTCGCGCATACAGCCGG
GCAGTGACGTCATCGTCTGCGCGGAAATGGACGAACAGTGGGGCTATGTCGGGGCTAAATCGCGCCAGCGCTGGCTGTTTTACG
CGTATGACAGTCTCCGGAAGACGGTTGTTGCGCACGTATTCGGTGAACGCACTATGGCGACGCTGGGGCGTCTTATGAGCCTGCT
GTCACCCTTTGACGTGGTGATATGGATGACGGATGGCTGGCCGCTGTATGAATCCCGCCTGAAGGGAAAGCTGCACGTAATCAGC
AAGCGATATACGCAGCGAATTGAGCGGCATAACCTGAATCTGAGGCAGCACCTGGCACGGCTGGGACGGAAGTCGCTGTCGTTC
TCAAAATCGGTGGAGCTGCATGACAAAGTCATCGGGCATTATCTGAACATAAAACACTATCAATAAGTTGGAGTCATTACCCAACC
AGGAAGGGCAGCCCACCTATCAAGGTGTACTGCCTTCCAGACGAACGAAGAGCGATTGAGGAAAAGGCGGCGGCGGCCGGCAT
GAGCCTGTCGGCCTACCTGCTGGCCGTCGGCCAGGGCTACAAAATCACGGGCGTCGTGGACTATGAGCACGTCCGCGAGCTGGC
CCGCATCAATGGCGACCTGGGCCGCCTGGGCGGCCTGCTGAAACTCTGGCTCACCGACGACCCGCGCACGGCGCGGTTCGGTGA
TGCCACGATCCTCGCCCTGCTGGCGAAGATCGAAGAGAAGCAGGACGAGCTTGGCAAGGTCATGATGGGCGTGGTCCGCCCGAG
GGCAGAGCCATGACTTTTTAGCCGCTAAAACGGCCGGGGGGTGCGCGTGATTGCCAAGCACGTCCCCATGCGCTCCATCAAGAA
GAGCGACTTCGCGGAGCTGGTATTCGTGCAGGGCAAGATTCGGAATACCAAGTACGAGAAGGACGGCCAGACGGTCTACGGGA
CCGACTTCATTGCCGATAAGGTGGATTATCTGGACACCAAGGCACCAGGCGGGTCAAATCAGGAATAAGGGCACATTGCCCCGG
CGTGAGTCGGGGCAATCCCGCAAGGAGGGTGAATGAATCGGACGTTTGACCGGAAGGCATACAGGCAAGAACTGATCGACGCG
GGGTTTTCCGCCGAGGATGCCGAAACCATCGCAAGCCGCACCGTCATGCGTGCGCCCCGCGAAACCTTCCAGTCCGTCGGCTCGA
TGGTCCAGCAAGCTACGGCCAAGATCGAGCGCGACAGCGTGCAACTGGCTCCCCCTGCCCTGCCCGCGCCATCGGCCGCCGTGGA
GCGTTCGCGTCGTCTCGAACAGGAGGCGGCAGGTTTGGCGAAGTCGATGACCATCGACACGCGAGGAACTATGACGACCAAGAA
GCGAAAAACCGCCGGCGAGGACCTGGCAAAACAGGTCAGCGAGGCCAAGCAGGCCGCGTTGCTGAAACACACGAAGCAGCAGA
TCAAGGAAATGCAGCTTTCCTTGTTCGATATTGCGCCGTGGCCGGACACGATGCGAGCGATGCCAAACGACACGGCCCGCTCTGC
CCTGTTCACCACGCGCAACAAGAAAATCCCGCGCGAGGCGCTGCAAAACAAGGTCATTTTCCACGTCAACAAGGACGTGAAGATC
ACCTACACCGGCGTCGAGCTGCGGGCCGACGATGACGAACTGGTGTGGCAGCAGGTGTTGGAGTACGCGAAGCGCACCCCTATC
GGCGAGCCGATCACCTTCACGTTCTACGAGCTTTGCCAGGACCTGGGCTGGTCGATCAATGGCCGGTATTACACGAAGGCCGAGG
AATGCCTGTCGCGCCTACAGGCGACGGCGATGGGCTTCACGTCCGACCGCGTTGGGCACCTGGAATCGGTGTCGCTGCTGCACCG
CTTCCGCGTCCTGGACCGTGGCAAGAAAACGTCCCGTTGCCAGGTCCTGATCGACGAGGAAATCGTCGTGCTGTTTGCTGGCGAC
CACTACACGAAATTCATATGGGAGAAGTACCGCAAGCTGTCGCCGACGGCCCGACGGATGTTCGACTATTTCAGCTCGCACCGGG
AGCCGTACCCGCTCAAGCTGGAAACCTTCCGCCTCATGTGCGGATCGGATTCCACCCGCGTGAAGAAGTGGCGCGAGCAGGTCG
GCGAAGCCTGCGAAGAGTTGCGAGGCAGCGGCCTGGTGGAACACGCCTGGGTCAATGATGACCTGGTGCATTGCAAACGCTAG
GGCCTTGTGGGGTCAGTTCCGGCTGGGGGTTCAGCAGCCAGCGCTTTACTGGCATTTCAGGAACAAGCGGGCACTGCTCGACGC
ACTTGCTTCGCTCAGTATCGCTCGGGACGCACGGCGCGCTCTACGAACTGCCGATAAACAGAGGATTAAAATTGACAATTGTGATT
AAGGCTCAGATTCGACGGCTTGGAGCGGCCGACGTGCAGGATTTCCGCGAGATCCGATTGTCGGCCCTGAAGAAAGCTCCAGAG
ATGTTCGGGTCCGTTTACGAGCACGAGGAGAAAAAGCCCATGGAGGCGTTCGCTGAACGGTTGCGAGATGCCGTGGCATTCGGC
GCCTACATCGACGGCGAGATCATTGGGCTGTCGGTCTTCAAACAGGAGGACGGCCCCAAGGACGCTCACAAGGCGCATCTGTCC
GGCGTTTTCGTGGAGCCCGAACAGCGAGGCCGAGGGGTCGCCGGTATGCTGCTGCGGGCGTTGCCGGCGGGTTTATTGCTCGTG
ATGATCGTCCGACAGATTCCAACGGGAATCTGGTGGATGCGCATCTTCATCCTCGGCGCACTTAATATTTCGCTATTCTGGAGCTT
GTTGTTTATTTCGGTCTACCGCCTGCCGGGCGGGGTCGCGGCGACGGTAGGCGCTGTGCAGCCGCTGATGGTCGTGTTCATCTCT
GCCGCTCTGCTAGGTAGCCCGATACGATTGATGGCGGTCCTGGGGGCTATTTGCGGAACTGCGGGCGTGGCGCTGTTGGTGTTG
ACACCAAACGCAGCGCTAGATCCTGTCGGCGTCGCAGCGGGCCTGGCGGGGGCGGTTTCCATGGCGTTCGGAACCGTGCTGACC
CGCAAGTGGCAACCTCCCGTGCCTCTGCTCACCTTTACCGCCTGGCAACTGGCGGCCGGAGGACTTCTGCTCGTTCCAGTAGCTTT
AGTGTTTGATCCGCCAATCCCGATGCCTACAGGAACCAATGTTCTCGGCCTGGCGTGGCTCGGCCTGATCGGAGCGGGTTTAACC
TACTTCCTTTGGTTCCGGGGGATCTCGCGACTCGAACCTACAGTTGTTTCCTTACTGGGCTTTCTCAGCCGGGATGGCGCTAAGAA

GCTATTGCCGCCGATCTTCATATGCGGTGTGAAATACCGCACAGATGCGTAAGGAGAAAATACCGCATCAGGCGCTCTTCCGCTTC
CTCGCTCACTGACTCGCTGCGCTCGGTCGTTCGGCTGCGGCGAGCGGTATCAGCTCACTCAAAGGCGGTAATACGGTTATCCACA
GAATCAGGGGATAACGCAGGAAAGAACATGTGAGCAAAAGGCCAGCAAAAGGCCAGGAACCGTAAAAAGGCCGCGTTGCTGGC
GTTTTTCCATAGGCTCCGCCCCCCTGACGAGCATCACAAAAATCGACGCTCAAGTCAGAGGTGGCGAAACCCGACAGGACTATAA
AGATACCAGGCGTTTCCCCCTGGAAGCTCCCTCGTGCGCTCTCCTGTTCCGACCCTGCCGCTTACCGGATACCTGTCCGCCTTTCTC
CCTTCGGGAAGCGTGGCGCTTTCTCAATGCTCACGCTGTAGGTATCTCAGTTCGGTGTAGGTCGTTCGCTCCAAGCTGGGCTGTGT
GCACGAACCCCCCGTTCAGCCCGACCGCTGCGCCTTATCCGGTAACTATCGTCTTGAGTCCAACCCGGTAAGACACGACTTATCGC
CACTGGCAGCAGCCACTGGTAACAGGATTAGCAGAGCGAGGTATGTAGGCGGTGCTACAGAGTTCTTGAAGTGGTGGCCTAACT
ACGGCTACACTAGAAGGACAGTATTTGGTATCTGCGCTCTGCTGAAGCCAGTTACCTTCGGAAAAAGAGTTGGTAGCTCTTGATCC
GGCAAACAAACCACCGCTGGTAGCGGTGGTTTTTTTGTTTGCAAGCAGCAGATTACGCGCAGAAAAAAAGGATATCAAGAAGATC
CTTTGATCTTTTCTACGGGGTCTGACGCTCAGTGGAACGAAAACTCACGTTAAGGGATTTTGGTCATGAGATTATCAAAAAGGATC
TTCACCTAGATCCTTTTAAATTAAAAATGAAGTTTTAAATCAATCTAAAGTATATATGAGTAAACTTGGTCTGACAGTTACCAATGC
TTAATCAGTGAGGCACCTATCTCAGCGATCTGTCTATTTCGTTCATCCATAGTTGCCTGACTCCCCGTCGTGTAGATAACTACGATA
CGGGAGGGCTTACCATCTGGCCCCAGTGCTGCAATGATACCGCGAGACCCACGCTCACCGGCTCCAGATTTATCAGCAATAAACC
AGCCAGCCGGAAGGGCCGAGCGCAGAAGTGGTCCTGCAACTTTATCCGCCTCCATCCAGTCTATTAAACAAGTGGCAGCAACGGA
TTCGCAAACCTGTCACGCCTTTTGTGCCAAAAGCCGCGCCAGGTTTGCGATCCGCTGTGCCAGGCGTTAGGCGTCATATGAAGATT
TCGGTGATCCCTGAGCAGGTGGCGGAAACATTGGATGCTGAGAACCATTTCATTGTTCGTGAAGTGTTCGATGTGCACCTATCCG
ACCAAGGCTTTGAACTATCTACCAGAAGTGTGAGCCCCTACCGGAAGGATTACATCTCGGATGATGACTCTGATGAAGACTCTGCT
TGCTATGGCGCATTCATCGACCAAGAGCTTGTCGGGAAGATTGAACTCAACTCAACATGGAACGATCTAGCCTCTATCGAACACAT
TGTTGTGTCGCACACGCACCGAGGCAAAGGAGTCGCGCACAGTCTCATCGAATTTGCGAAAAAGTGGGCACTAAGCAGACAGCT
CCTTGGCATACGATTAGAGACACAAACGAACAATGTACCTGCCTGCAATTTGTACGCAAAATGTGGCTTTACTCTCGGCGGCATTG
ACCTGTTCACGTATAAAACTAGACCTCAAGTCTCGAACGAAACAGCGATGTACTGGTACTGGTTCTCGGGAGCACAGGATGACGC
CTAACAATTCATTCAAGCCGACACCGCTTCGCGGCGCGGCTTAATTCAGGAGTTAAACATCATGAGGGAAGCGGTGATCGCCGAA
GTATCGACTCAACTATCAGAGGTAGTTGGCGTCATCGAGCGCCATCTCGAACCGACGTTGCTGGCCGTACATTTGTACGGCTCCGC
AGTGGATGGCGGCCTGAAGCCACACAGTGATATTGATTTGCTGGTTACGGTGACCGTAAGGCTTGATGAAACAACGCGGCGAGC
TTTGATCAACGACCTTTTGGAAACTTCGGCTTCCCCTGGAGAGAGCGAGATTCTCCGCGCTGTAGAAGTCACCATTGTTGTGCACG
ACGACATCATTCCGTGGCGTTATCCAGCTAAGCGCGAACTGCAATTTGGAGAATGGCAGCGCAATGACATTCTTGCAGGTATCTTC
GAGCCAGCCACGATCGACATTGATCTGGCTATCTTGCTGACAAAAGCAAGAGAACATAGCGTTGCCTTGGTAGGTCCAGCGGCGG
AGGAACTCTTTGATCCGGTTCCTGAACAGGATCTATTTGAGGCGCTAAATGAAACCTTAACGCTATGGAACTCGCCGCCCGACTGG
GCTGGCGATGAGCGAAATGTAGTGCTTACGTTGTCCCGCATTTGGTACAGCGCAGTAACCGGCAAAATCGCGCCGAAGGATGTC
GCTGCCGACTGGGCAATGGAGCGCCTGCCGGCCCAGTATCAGCCCGTCATACTTGAAGCTAGGCAGGCTTATCTTGGACAAGAAG
ATCGCTTGGCCTCGCGCGCAGATCAGTTGGAAGAATTTGTTCACTACGTGAAAGGCGAGATCACCAAGGTAGTCGGCAAATAATG
TCTAACAATTCGTTCAAGCCGACGCCGCTTCGCGGCGCGGCTTAACTCAAGCGTTAGAGAGCTGGGGAAGACTATGCGCGATCTG
TTGAAGGTGGTTCTAAGCCTCGTACTTGCGATGGCATCGGGGCAGGCACTTGCTGACCTGCCAATTGTTTTAGTGGATGAAGCTC
GTCTTCCCTATGACTACTCCCCATCCAACTACGACATTTCTCCAAGCAACTACGACAACTCCATAAGCAATTACGACAATAGTCCAT
CAAATTACGACAACTCTGAGAGCAACTACGATAATAGTTCATCCAATTACGACAATAGTCGCAACGGAAATCGTAGGCTTATATAT
AGCGCAAATGGGTCTCGCACTTTCGCCGGCTACTACGTCATTGCCAACAATGGGACAACGAACTTCTTTTCCACATCTGGCAAAAG
GATGTTCTACACCCCAAAAGGGGGGCGCGGCGTCTATGGCGGCAAAGATGGGAGCTTCTGCGGGGCATTGGTCGTCATAAATGG
CCAATTTTCGCTTGCCCTGACAGATAACGGCCTGAAGATCATGTATCTAAGCAACTAGCCTGCTCTCTAATAAAATGTTAGGAGCTT
GGCTGCCATTTTTGGGGTGAGGCCGTTCGCGGCCGAGGGGCGCAGCCCCTGGGGGGATGGGAGGCCCGCGTTAGCGGGCCGGG
AGGGTTCGAGAAGGGGGGGCACCCCCCTTCGGCGTGCGCGGTCACGCGCCAGGGCGCAGCCCTGGTTAAAAACAAGGTTTATAA
ATATTGGTTTAAAAGCAGGTTAAAAGACAGGTTAGCGGTGGCCGAAAAACGGGCGGAAACCCTTGCAAATGCTGGATTTTCTGCC
TGTGGACAGCCCCTCAAATGTCAATAGGTGCGCCCCTCATCTGTCAGCACTCTGCCCCTCAAGTGTCAAGGATCGCGCCCCTCATC
TGTCAGTAGTCGCGCCCCTCAAGTGTCAATACCGCAGGGCACTTATCCCCAGGCTTGTCCACATCATCTGTGGGAAACTCGCGTAA
AATCAGGCGTTTTCGCCGATTTGCGAGGCTGGCCAGCTCCACGTCGCCGGCCGAAATCGAGCCTGCCCCTCATCTGTCAACGCCGC
GCCGGGTGAGTCGGCCCCTCAAGTGTCAACGTCCGCCCCTCATCTGTCAGTGAGGGCCAAGTTTTCCGCGAGGTATCCACAACGC
CGGCGGCCGGCCGCGGTGTCTCGCACACGGCTTCGACGGCGTTTCTGGCGCGTTTGCAGGGCCATAGACGGCCGCCAGCCCAGC
GGCGAGGGCAACCAGCCCGGTGAGCGTCGGAAAGGGTCGACATCTTGCTGCGTTCGGATATTTTCGTGGAGTTCCCGCCACAGA
CCCGGATTGAAGGCGAGATCCAGCAACTCGCGCCAGATCATCCTGTGACGGAACTTTGGCGCGTGATGACTGGCCAGGACGTCG
GCCGAAAGAGCGACAAGCAGATCACGATTTTCGACAGCGTCGGATTTGCGATCGAGGATTTTTCGGCGCTGCGCTACGTCCGCGA
CCGCGTTGAGGGATCAAGCCACAGCAGCCCACTCGACCTTCTAGCCGACCCAGACGAGCCAAGGGATCTTTTTGGAATGCTGCTC

CGTCGTCAGGCTTTCCGACGTTTGGGTGGTTGAACAGAAGTCATTATCGTACGGAATGCCAGCACTCCCGAGGGGAACCCTGTGG
TTGGCATGCACATACAAATGGACGAACGGATAAACCTTTTCACGCCCTTTTAAATATCCGTTATTCTAATAAACGCTCTTTTCTCTTA
GGTTTACCCGCCAATATATCCTGTCAAACACTGATAGTTTAAACTGAAGGCGGGAAACGACAATCTGATCATGAGCGGAGAATTA
AGGGAGTCACGTTATGACCCCCGCCGATGACGCGGGACAAGCCGTTTTACGTTTGGAACTGACAGAACCGCAACGATTGAAGGA
GCCACTCAGCCCCAATACGCAAACCGCCTCTCCCCGCGCGTTGGCCGATTCATTAATGCAGCTGGCACGACAGGTTTCCCGACTGG
AAAGCGGGCAGTGAGCGCAACGCAATTAATGTGAGTTAGCTCACTCATTAGGCACCCCAGGCTTTACACTTTATGCTTCCGGCTCG
TATGTTGTGTGGAATTGTGAGCGGATAACAATTTCACACAGGAAACAGCTATGACCATGATTACGCCAAGCTATTTAGGTGACACT
ATAGAATACTCAAGCTATGCATCCAACGCGTTGGGAGCTCCCTAGG CTTTTTTTCTTCTTCTTCGTTCATACAGTTTTTTTTTGTTTAT
CAGCTTACATTTTCTTGAACCGTAGCTTTCGTTTTCTTCTTTTTAACTTTCCATTCGGAGTTTTTGTATCTTGTTTCATAGTTTGTCCCA
GGATTAGAATGATTAGGCATCGAACCTTCAAGAATTTGATTGAATAAAACATCTTCATTCTTAAGATATGAAGATAATCTTCAAAA
GGCCCCTGGGAATCTGAAAGAAGAGAAGCAGGCCCATTTATATGGGAAAGAACAATAGTATTTCTTATATAGGCCCATTTAAGTT
GAAAACAATCTTCAAAAGTCCCACATCGCTTAGATAAGAAAACGAAGCTGAGTTTATATACAGCTAGAGTCGAAGTAGTGATT GA
ATGTCTGTTGCCTTGTTAGTTTTAGAGCTAGAAATAGCAAGTTAAAATAAGGCTAGTCCGTTATCAACTTGAAAAAGTGGCACCGA
GTCGGTGCTTTTTTTCTAGACCCAGCTTTCTTGTACAAAGTTGGCATT

Plasmids for Allele-specific DSB induction and allele dependent repair:

We used similar plasmids as for Tomato fruit color assay with different gRNA molecule:

GAGCGTATATAATGCTGCTTGTTTTAGAGCTAGAAATAGCAAGTTAAAATAAGGCTAGTCCGTTATCAACTTGAAAAAGTGGCACC
GAGTCGGTGCTTTTTTT

Plasmids for synthetic crossover control (inverse PCR assay):

>R1

GGAGCAACCTTATTTTGTACTTTAAAAAATTCATTTTTTTTATTTTTTCGACTTTAAAGCCAAAATTATCCTTTATTTATGAAAGTGGA
TGTATTTTTATCCCTTTAATATAAGGTTGAGTTTTCTTTAAGTTTGTAATGTTAAGTGGGGCACTTTATATTAGTCCAATTA ACAGAA
TTGACTTAGAAATTGATGGCGATTACTTGAAAAAATCGCTACAAAAACATAATAAAATAAAATTTCATTTGATTTTTAAACCAAAAG
AAAAGTGACATGACTAACGATTTGATCCCATCTTCTTTTTTTTTTAAGAAAAAAATTAAAAGAAAAATTTATTAAACTGGATGATTA
ATTTTAACATAAAAATTAAAAAAATAAAAATCAATCGAGCTCACTATTTGATATTATTATTTTCATCTTGATATTG TTACTTCATATCA
CTGAATTATAAAATGCGTTAACGGCAATTATCATAGACGCAGACTGAAAGCATAAAATTACAACTATTCCATTTTTGTTTTTTTTTTG
CCAACCCAAAAAAAACTAATAATTTATTTACATAATTATAGAAAATTGGAATTTATTCATGTTTTTGGACCATTCAGATAACCCCAC
AATAAAAAAAAAGTGCACAAAAGTGCAAAGTGCTACGTGTCCTAATTATAGCCATACTACATTTGTGCATAAAATTAGCCACCATT
ATTTTTAAGATTATTCTTTTTCTTGTTTTAATTTATATGCCACACTTTCTTATTAATCCATCTTAAACAGAATGACACACTTTTTATTTG
ACAAATATTTAATGACATTATTAACATTTTATCCTCGTTAGATTTCACTTATTTGGTTAAAGGTGGTGTACTTTTCTAATCTAAAATC
ATTAATTTTAGGTATAATTTTAGAACATTGCAAAGCCTTTTCATATATTTAAACAATGTA CATAATTATGTAATTATGTTATGTTATAT
AAATTGAGACGAAAAAAATATTATTTATCAGGTACGAGTGTCACTTTACACTAGAGGTGTTTACGGACAGAGACGAATCCAAGAT
TTGAACTTCATGGTGTCAAGTATACACATTGCTTAGATTATTAAATCAACTTTAGGTTTCAATTCCGCACAAAATCTATAAAACATA
ACCTTTTTAAAAATAATTTTACTAATATTATAAACATATATTATTCATTTATAAT AGTTCTCGACGAGTTTACATATTTTCAAAACTGT
CAATTAGAACATCATTACTTATATTTGTAAATGTATCACTTACTACGTAACAAACTAAACAAATCATTTGAATATCACTCATCACTCT
TATTTATATTTTATTTTTTTATCTACTTCACCAAAGAAAATGATAATTTCACAATTGCGATTGCTACCAGAAAAATATTTACTTAATCA
ATTTTAAAAAGATTAAGTAATAGAAATATTTTTTATTTTATTAAGAAAAGAGGATTTTATCATATCTAAATATAAATTAAAGATGAA
GTTACTATGATATTATTTAAAAAGTAAATGACATTATTCTATTTAAATTACAAAATGAGATTAAGAAAAGTATAACTATTTCCATTTT
TTTAGCTAAAAGGATTTCAAAAAAAAAAGTAACAAGAAAGAGATTTTTTAAAAATAATAATTTTATTAGAAATATATATAAAAAAT
TAATAATATTCGTTAGAAAAAGTGAATTTTCAAAATAATAAAGTAATAACACGAATACTTTTAGGGTCAAACAATTAATAGTGGAG
AATGGAGATATTTTTAGTACAAATTATTTATACAAAATATCTTTATTCAATGTGACATAATTTTAAGCTATTTTTAACACTTTTCCATC
AAATAAATAATTAATAACAAATTATTAAAAAATCAAAGATTAATAAATTAAATTAATGAACTATAATTAGTTATTAAAACCAAGCCA
ACTAGAATTAAATAGAAAAAAGGAAATATAATCTTCAAAATATGCTTTGTTGAAGTATAATTCCTCGTCTATTTAAAGCTTCGGAG
ATTTAAATTGGCAAGCGAATTACACAAAGCAAATGAAACTACACCTTTTGGTTCTGTGGGTGTCATTTGCTTATTTATAAACATTGT
ACATATATACACCTATATGTATATCACGTTTCGATCGAAGCTCGTGGGTGGCGTGGCACCCATAAATCGTAGTAGATTCGCCCCTG

TTTACGGATCGATTTAGGTCGGTTATAGATCAAAATCAAATCAATTTAATTAATTTTACCAATCAAACCAAATCAAATATACTATAC
ATTTATTGGTTTGGTTGTTCGAATTTCGATTTGATTTGATTCAATTATTCGATTATCAATCATACAGAAATAAAAGAAACAATTCATT
TTAAAGAGGAACAAAAGAGGCAAGAACTCATTCAAAATGAAAAATACTTAAAATAGCCGAATTTATAAAACTTTTTATTACTAAAT
TTACTGTGAATAAAACTTTTAAATTCACCATTTTAGCCTAGAAGTAAGAGAGAAAAACATTTCCACCAAGAATTGTCAAGAATACTC
ATATACATGAAACCAATATGATAAATGTTCTCTTCAGGACGGTTCATAAATAAATTATGATAAACATATATAAGATATTTAAAATAA
AATAAAATATATATATATAAAATTTATCGGTTCGATTATTTCTTCAACTTTTTAAATAAAATCAAAGACTATCAATTCTTAAGAATCT
TAGAACAAACTAAACCAAATAAAATTAAACGATTTAATCAACTTAATTTAATTTTTCAATGTGGGTCAATTTTTATCCAAATACTTTT
ACACCCACTTTTCACCATCACAAGGGGTAAGAACTAGCAAATTATTTTGCCTTAACAAAAACTTACTATTGGTGTAGAGAAAATTG
GTTGATTTTCTTATCAATATATATAATTTAGTACCCAATTTTCCCAAAACTAAATAAAATATAGAATATCCACTAAGGTGGTTACACG
TGTACACTCAACCAATGACGGCACTTCAAATTCTTGATAACGGTATCTTCCCACCTTCATATATTTCAACATTTTATTGTTAAAATAA
AATCGCACGCTCTATTTTATCTAAATTTTATTTATAGAATTATATTAAGTATGTTATTATTAATTTTTACGTATAAATATATTTCATATT
AAAATCATATAAGTTGACATCACTAAGTTCGTTGGATTTGCATGTAGACCCAATTATTCAGATACTCAACATGACTCATTTAATATT
GGATTATTGAAATATTTGTAATACATTAAGAATATAATTGTTAACTATTTTAATTTTTAATTATACGTAAATATCAAACAAAAATATT
AATATGATCGCTATATTAGATGATAACTATAAGGAGCCTACACAATTAACACTATTTAACTCTATTCTTTGCATTTATAAAAAGTTAC
TTTAGTCTTAGGTTCACAATGTCAAAATCTAAACAACTAAAAACGACGAGGAGTAAGGTTTGCAACGACGATAACAAGGATTAGG
CAACAATTAGAGTTGTGAATTGTGAGTATTAACTATACTTTTACTATATTAGGCAGAATTTTTGCACTCAATGAGTAACTTGATTTAT
TTATTTTTTATTTCGCCCTAAATTATTGGACAAGTCATATATTTGTTTTGAAAACATTCTTTTATTGGCTAAATCGAAAATTGAATCGT
TAAAGATCAAAAATCAATAACAAATATCTTATTGGTTTAACATATTTAAAAATAAAAAACCAATAAATCTAACTAATAA TATTTAAT
ACGAAAACGAAATGGACTGACACACATTCCTAAATTTTTGGTCAAAATTTTTTCATAATTTCCCTAAAATCTAAAATATTAAATATTT
GACGGAAACAAAAAATTCACTTTTAATAAATTATTTGAAGGACTAAAACAGTGGAAGAATATATTTAAGAAGCTAATTTGAACCTA
GTGCCAAATATAAAGGGACCATTTTTGTCATTTTTCAACTTGAAAATCTACGTGTCTTAATATAACACCAAAGA ATTAATATTTACT
GAAAAAATGTAAAAATGAGGATATGGATTCTGAATCACTCAATTCCAATCAGCAAAAATAAAATAAAATAAAATAAAATAAAATTT
AAAAAATAATAATAAATGCTATAAAATGACCAAAATGTGTGGAGCAAAAAGTGCAGAAAAAACCAACAAATTGCATTCTCCATTC
TTGGAAGTGGCCATTCTTGATTTCTTGAAACAAAGGTTTGTTTCCCTTCACTTCTTGATATGTAAAGTTGCA ATCTTTATAACTTTCT
ATTGCTTTGCTAGTGTTTTTGTTATATACAGGGGGTGGAGTTAGAGGGTAAGTTACGCATTTAGTCGTAACTTTAGTCAAACTTCGT
AATAATTTAGTAAGTTAAAATATATTAGAAATTTTCAGAATTCATAAACTTTAAATTTTAAATTTTGACTTCGCTTTGTGTGACTATA
CAATTACAGAAATTCAGAGTGGCCATTGTTGAAAGAGAGGGTGGAATTTGTGTAAGTTTTGTTTC CTTTCAGTTCTTGATATATAA
AGTTGCAATCTTTAACATTCTTTGTTCACTTTCTATAGGTTTGCTAGGTTCGGTTAAATTCAGTAGCTTTAGTTTAAACCCTATGCGG
AATAGAGAATGTGTAAACTTTAAACTTCAAATTTTGGCTCCGCATACGACTAGCGACTATATAATAATAGGAATTGAGCACTTGGC
TTTTGTATATAGCTTCTATGTGTACCAAAATTAGAAAATCAGGCGATTATTATAATCTTGTTGACTAAATATAGAATGCATCCATTAC
CCCCAAAAAGTGTGATTCCACTGTCATAGGAGGTTTTTTTTATTTCATTTTATTTGTGCTTTCAATAATGTAGAGTAGTTTTACAAAG
ATCCTTTCTTTGTGACACATGGTAGGTAATATTGCTGATTTTGTTGTAGTTTTGGGGTTATAAAGTTTCAAATTATTTATACTGGAG
GGTAGGGGTGGGGGTTGTCTATAATGCAGGTTATGGTTTTACGTGAACTCAATAATTATTGTAGATACTAAGAAATCCACTCAGT
GTTCTTGCGGTGTCTTGCTTTTGATTTCAGCATCACTTGTAGTTGATTGTGTTTAGATTATCACATTATTCTGTGGCTGTAACTGTAT
CCTTGTTAGTTGCTTTGTTTCTACACTGTTGTTTTCCCTCTTTTATACCTATTTTGATATGTTGTACTCGAACGAGGGTCATCGGGGA
ACAACCTCTTTACCTCCGTGAGGTAGAGCTATGGTCTGTGTCCACTCTACCCTCCCCAGATCCCTCTTGTAGGATTTCACTATATTGT
AATATTAACTTGAGGTCACTATAGGAGCTCAAAAACTTCTAATTTTGAATCAATGTCTGGTTATACTTTTTTTGTCATAACTGTATCT
CAAATGTGGTGTTTGGTTTATCTCATTTTGCAGAAGTCAAGAAACAGGTTACTCCTGTTTGAGTGAGGAAAAGTTGGTTTGCCTGT
CTGTGGTCTTTTTATAATCTTTTTCTACAGAAGAGAAAGTGGGTAATTTTGTTTGAGAGTGGAAATATTCTCTAGTGGGAATCTACT
AGGAGTAATTTATTTTCTATAAACTAAGTAAAGTTTGGAAGGTGACAAAAAGAAAGACAAAAATCTTGGAATTGTTTTAGACAACC
AAGGTTTTCTTGCTCAGAATGTCTGTTGCCTTGTTATGGGTTGTTTCTCCTTGTGACGTCTCAAATGGGACAAGTTTCATGGAATCA
GTCCGGGAGGGAAACCGTTTTTTTGATTCATCGAGGCATAGGAATTTGGTGTCCAATGAGAGAATCAATAGAGGTGGTGGAAAG
CAAACTAATAATGGACGGAAATTTTCTGTACGGTCTGCTATTTTGGCTACTCCATCTGGAGAACGGACGATGACATCGGAACAGAT
GGTCTATGATGTGGTTTTGAGGCAGGCAGCCTTGGTGAAGAGGCAACTGAGATCTACCAATGAGTTAGAAGTGAAGCCGGATAT
ACCTATTCCGGGGAATTTGGGCTTGTTGAGTGAAGCATATGATAGGTGTGGTGAAGTATGTGCAGAGTATGCAAAGACGTTTAAC
TTAGGTTAGCTTCTTCAATCTATTCATTCGTTTACCAAATATTATTTGGTAAGCACTAATTATGAATATATATATGTTCATGTTATTGA
TGAAGACAAAATTTGATCTTTGTTTGTTTATTCAGGAACTATGCTAATGACTCCCGAGAGAAGAAGGGCTATCTGGGCAATATATG
GTGAGGTTTCTAGCCATTTAATAACAGTTACGCGCACAAACACATATGATTAATCGGGGACGAGAAAAAAAGAAATGAAGTTTGA
GTTTTGAGGGTCATATGTAATAGGTAAATCCGAGCTTGACTAGCTTGAGATGTTTATTGTCATATCATGCTCAATACTAACCAAAAC
ACTGAAAAAGAACTTGATTATATTTACATACTAATATTTTCATTTGCGTTGCTGTTCACATTTTTACCTATGGAACTGGTTTTTGTGA
TTTGTTATACTTCATATTCGATGTTAATAAAATATATCATTCCTCCCTTTTTCTCCACTTCAAGCTTTACTGTAGTGTTGAAAGGGGA

AACTCCTTTTAATGATTGCATATATAAACGAACTTCTTGAGTTGAATAGTTTCTCATTATGATCTGTTTAAACAGTATGGTGCAGAA
GAACAGATGAACTTGTTGATGGCCCAAACGCATCATATATTACCCCGGCAGCCTTAGATAGGTGGGAAAATAGGCTAGAAGATGT
TTTCAATGGGCGGCCATTTGACATGCTCGATGGTGCTTTGTCCGATACAGTTTCTAACTTTCCAGTTGATATTCAGGTTAGTCTACC
AATTCTATGGTCTTTATATTTGTTCAATTTGCGTTTGATGTCACTTTTGCTGAGGGCTTTTCTAATAGCTTACTTCAGCCTAGCGGAA
ATGTTTGTAGTTGAATCTCTAGTTCTGTCTCCTATATCTGTTTCTCTCGTCCTAGATACTACACATACTTCATTTCTGTTTTAACATTTT
ATTCGTCTTTTGGTGTTGTTTTGTATGTGAATCATATATTTGGAACAGAATCATTATTAGTTCACATGATTTCATTTGCTTTCTTCAAT
AGCGTAATTGTCTAACCTTCCAATATATGTTGCAGCCATTCAGAGATATGATTGAAGGAATGCGTATGGACTTGAGAAAATCGAG
ATACAAAAACTTCGACGAACTATACCTTTATTGTTATTATGTTGCTGGTACGGTTGGGTTGATGAGTGTTCCAATTATGGCGCTGTC
ATGAGACGAATTCTGACAGGATATATTGGCGGGTAAACCTAAGAGAAAAGAGCGTTTATTAGAATAATCGGATATTTAAAAGGG
CGTGAAAAGGTTTATCCGTTCGTCCATTTGTATGTGCATGCCAACCACAGGGTTCCCCTCGGGATCAAAGTACTTTGATCCAACCCC
TCCGCTGCTATAGTGCAGTCGGCTTCTGACGTTCAGTGCAGCCGTCATCTGAAAACGACATGTCGCACAAGTCCTAAGTTACGCGA
CAGGCTGCCGCCCTGCCCTTTTCCTGGCGTTTTCTTGTCGCGTGTTTTAGTCGCATAAAGTAGAATACTTGCGACTAGAACCGGAG
ACATTACGCCATGAACAAGAGCGCCGCCGCTGGCCTGCTGGGCTATGCCCGCGTCAGCACCGACGACCAGGACTTGACCAACCAA
CGGGCCGAACTGCACGCGGCCGGCTGCACCAAGCTGTTTTCCGAGAAGATCACCGGCACCAGGCGCGACCGCCCGGAGCTGGCC
AGGATGCTTGACCACCTACGCCCTGGCGACGTTGTGACAGTGACCAGGCTAGACCGCCTGGCCCGCAGCACCCGCGACCTACTGG
ACATTGCCGAGCGCATCCAGGAGGCCGGCGCGGGCCTGCGTAGCCTGGCAGAGCCGTGGGCCGACACCACCACGCCGGCCGGC
CGCATGGTGTTGACCGTGTTCGCCGGCATTGCCGAGTTCGAGCGTTCCCTAATCATCGACCGCACCCGGAGCGGGCGCGAGGCCG
CCAAGGCCCGAGGCGTGAAGTTTGGCCCCCGCCCTACCCTCACCCCGGCACAGATCGCGCACGCCCGCGAGCTGATCGACCAGGA
AGGCCGCACCGTGAAAGAGGCGGCTGCACTGCTTGGCGTGCATCGCTCGACCCTGTACCGCGCACTTGAGCGCAGCGAGGAAGT
GACGCCCACCGAGGCCAGGCGGCGCGGTGCCTTCCGTGAGGACGCATTGACCGAGGCCGACGCCCTGGCGGCCGCCGAGAATG
AACGCCAAGAGGAACAAGCATGAAACCGCACCAGGACGGCCAGGACGAACCGTTTTTCATTACCGAAGAGATCGAGGCGGAGAT
GATCGCGGCCGGGTACGTGTTCGAGCCGCCCGCGCACCTCTCAACCGTGCGGCTGCATGAAATCCTGGCCGGTTTGTCTGATGCC
AAGCTGGCGGCCTGGCCGGCCAGCTTGGCCGCTGAAGAAACCGAGCGCCGCCGTCTAAAAAGGTGATGTGTATTTGAGTAAAAC
AGCTTGCGTCATGCGGTCGCTGCGTATATGATCCGATGAGTAAATAAACAAATACGCAAGGGGAACGCATGAAGGTTATCGCTGT
ACTTAACCAGAAAGGCGGGTCAGGCAAGACGACCATCGGAACCCATCTAGCCCGCGCCCTGCAACTCGCCGGGGCCGATGTTCT
GTTAGTCGATTCCGATCCCCAGGGCAGTGCCCGCGATTGGGCGGCCGTGCGGGAAGATCAACCGCTAACCGTTGTCGGCATCGA
CCGCCCGACGATTGACCGCGACGTGAAGGCCATCGGCCGGCGCGACTTCGTAGTGATCGACGGAGCGCCCCAGGCGGCGGACTT
GGCTGTGTCCGCGATCAAGGCAGCCGACTTCGTGCTGATTCCGGTGCAGCCAAGCCCTTACGACATATGGGCCACCGCCGACCTG
GTGGAGCTGGTTAAGCAGCGCATTGAGGTCACGGATGGAAGGCTACAAGCGGCCTTTGTCGTGTCGCGGGCGATCAAAGGCACG
CGCATCGGCGGTGAGGTTGCCGAGGCGCTGGCCGGGTACGAGCTGCCCATTCTTGAGTCCCGTATCACGCAGCGCGTGAGCTAC
CCAGGCACTGCCGCCGCCGGCACAACCGTTCTTGAATCAGAACCCGAGGGCGACGCTGCCCGCGAGGTCCAGGCGCTGGCCGCT
GAAATTAAATCAAAACTCATTTGAGTTAATGAGGTAAAGAGAAAATGAGCAAAAGCACAAACACGCTAAGTGCCGGCCGTCCGA
GCGCACGCAGCAGCAAGGCTGCAACGTTGGCCAGCCTGGCAGACACGCCAGCCATGAAGCGGGTCAACTTTCAGTTGCCGGCGG
AGGATCACACCAAGCTGAAGATGTACGCGGTACGCCAAGGCAAGACCATTACCGAGCTGCTATCTGAATAGATCGCGCAGCTACC
AGAGTAAATGAGCAAATGAATAAATGAGTAGATGAATTTTAGCGGCTAAAGGAGGCGGCATGGAAAATCAAGAACAACCAGGC
ACCGACGCCGTGGAATGCCCCATGTGTGGAGGAACGGGCGGTTGGCCAGGCGTAAGCGGCTGGGTTGTCTGCCGGCCCTGCAAT
GGCACTGGAACCCCCAAGCCCGAGGAATCGGCGTGACGGTCGCAAACCATCCGGCCCGGTACAAATCGGCGCGGCGCTGGGTG
ATGACCTGGTGGAGAAGTTGAAGGCCGCGCAGGCCGCCCAGCGGCAACGCATCGAGGCAGAAGCACGCCCCGGTGAATCGTGG
CAAGCGGCCGCTGATCGAATCCGCAAAGAATCCCGGCAACCGCCGGCAGCCGGTGCGCCGTCGATTAGGAAGCCGCCCAAGGGC
GACGAGCAACCAGATTTTTTCGTTCCGATGCTCTATGACGTGGGCACCCGCGATAGTCGCAGCATCATGGACGTGGCCGTTTTCCG
TCTGTCGAAGCGTGACCGACGAGCTGGCGAGGTGATCCGCTACGAGCTTCCAGACGGGCACGTAGAGGTTTCCGCAGGGCCGGC
CGGCATGGCCAGTGTGTGGGATTACGACCTGGTACTGATGGCGGTTTCCCATCTAACCGAATCCATGAACCGATACCGGGAAGGG
AAGGGAGACAAGCCCGGCCGCGTGTTCCGTCCACACGTTGCGGACGTACTCAAGTTCTGCCGGCGAGCCGATGGCGGAAAGCAG
AAAGACGACCTGGTAGAAACCTGCATTCGGTTAAACACCACGCACGTTGCCATGCAGCGTACGAAGAAGGCCAAGAACGGCCGC
CTGGTGACGGTATCCGAGGGTGAAGCCTTGATTAGCCGCTACAAGATCGTAAAGAGCGAAACCGGGCGGCCGGAGTACATCGAG
ATCGAGCTAGCTGATTGGATGTACCGCGAGATCACAGAAGGCAAGAACCCGGACGTGCTGACGGTTCACCCCGATTACTTTTTGA
TCGATCCCGGCATCGGCCGTTTTCTCTACCGCCTGGCACGCCGCGCCGCAGGCAAGGCAGAAGCCAGATGGTTGTTCAAGACGAT
CTACGAACGCAGTGGCAGCGCCGGAGAGTTCAAGAAGTTCTGTTTCACCGTGCGCAAGCTGATCGGGTCAAATGACCTGCCGGA
GTACGATTTGAAGGAGGAGGCGGGGCAGGCTGGCCCGATCCTAGTCATGCGCTACCGCAACCTGATCGAGGGCGAAGCATCCGC
CGGTTCCTAATGTACGGAGCAGATGCTAGGGCAAATTGCCCTAGCAGGGGAAAAAGGTCGAAAAGGACTCTTTCCTGTGGATAG
CACGTACATTGGGAACCCAAAGCCGTACATTGGGAACCGGAACCCGTACATTGGGAACCCAAAGCCGTACATTGGGAACCGGTC

ACACATGTAAGTGACTGATATAAAAGAGAAAAAAGGCGATTTTTCCGCCTAAAACTCTTTAAAACTTATTAAAACTCTTAAAACCC
GCCTGGCCTGTGCATAACTGTCTGGCCAGCGCACAGCCGAAGAGCTGCAAAAAGCGCCTACCCTTCGGTCGCTGCGCTCCCTACG
CCCCGCCGCTTCGCGTCGGCCTATCGCGGCCGCTGGCCGCTCAAAAATGGCTGGCCTACGGCCAGGCAATCTACCAGGGCGCGGA
CAAGCCGCGCCGTCGCCACTCGACCGCCGGCGCCCACATCAAGGCACCCTGCCTCGCGCGTTTCGGTGATGACGGTGAAAACCTC
TGACACATGCAGCTCCCGGTGACGGTCACAGCTTGTCTGTAAGCGGATGCCGGGAGCAGACAAGCCCGTCAGGGCGCGTCAGCG
GGTGTTGGCGGGTGTCGGGGCGCAGCCATGACCCAGTCACGTAGCGATAGCGGAGTGTATACTGGCTTAACTATGCGGCATCAG
AGCAGATTGTACTGAGAGTGCACCATATGCGGTGTGAAATACCGCACAGATGCGTAAGGAGAAAATACCGCATCAGGCGCTCTTC
CGCTTCCTCGCTCACTGACTCGCTGCGCTCGGTCGTTCGGCTGCGGCGAGCGGTATCAGCTCACTCAAAGGCGGTAATACGGTTAT
CCACAGAATCAGGGGATAACGCAGGAAAGAACATGTGAGCAAAAGGCCAGCAAAAGGCCAGGAACCGTAAAAAGGCCGCGTTG
CTGGCGTTTTTCCATAGGCTCCGCCCCCCTGACGAGCATCACAAAAATCGACGCTCAAGTCAGAGGTGGCGAAACCCGACAGGAC
TATAAAGATACCAGGCGTTTCCCCCTGGAAGCTCCCTCGTGCGCTCTCCTGTTCCGACCCTGCCGCTTACCGGATACCTGTCCGCCT
TTCTCCCTTCGGGAAGCGTGGCGCTTTCTCATAGCTCACGCTGTAGGTATCTCAGTTCGGTGTAGGTCGTTCGCTCCAAGCTGGGC
TGTGTGCACGAACCCCCCGTTCAGCCCGACCGCTGCGCCTTATCCGGTAACTATCGTCTTGAGTCCAACCCGGTAAGACACGACTT
ATCGCCACTGGCAGCAGCCACTGGTAACAGGATTAGCAGAGCGAGGTATGTAGGCGGTGCTACAGAGTTCTTGAAGTGGTGGCC
TAACTACGGCTACACTAGAAGGACAGTATTTGGTATCTGCGCTCTGCTGAAGCCAGTTACCTTCGGAAAAAGAGTTGGTAGCTCTT
GATCCGGCAAACAAACCACCGCTGGTAGCGGTGGTTTTTTTGTTTGCAAGCAGCAGATTACGCGCAGAAAAAAAGGATCTCAAGA
AGATCCTTTGATCTTTTCTACGGGGTCTGACGCTCAGTGGAACGAAAACTCACGTTAAGGGATTTTGGTCATGCATTCTAGGTGAT
TAGAAAAACTCATCGAGCATCAAATGAAACTGCAATTTATTCATATCAGGATTATCAATACCATATTTTTGAAAAAGCCGTTTCTGT
AATGAAGGAGAAAACTCACCGAGGCAGTTCCATAGGATGGCAAGATCCTGGTATCGGTCTGCGATTCCGACTCGTCCAACATCAA
TACAACCTATTAATTTCCCCTCGTCAAAAATAAGGTTATCAAGTGAGAAATCACCATGAGTGACGACTGAATCCGGTGAGAATGGC
AAAAGTTTATGCATTTCTTTCCAGACTTGTTCAACAGGCCAGCCATTACGCTCGTCATCAAAATCACTCGCATCAACCAAACCGTTA
TTCATTCGTGATTGCGCCTGAGCGAGTCGAAATACGCGATCGCTGTTAAAAGGACAATTACAAACAGGAATCGAATGCAACCGGC
GCAGGAACACTGCCAGCGCATCAACAATATTTTCACCTGAATCAGGATATTCTTCTAATACCTGGAATGCTGTTTTCCCTGGGATCG
CAGTGGTGAGTAACCATGCATCATCAGGAGTACGGATAAAATGCTTGATGGTCGGAAGAGGCATAAATTCCGTCAGCCAGTTTAG
TCTGACCATCTCATCTGTAACATCATTGGCAACGCTACCTTTGCCATGTTTCAGAAACAACTCTGGCGCATCGGGCTTCCCATACAA
TCGGTAGATTGTCGCACCTGATTGCCCGACATTATCGCGAGCCCATTTATACCCATATAAATCAGCATCCATGTTGGAATTTAATCG
CGGCCTTGAGCAAGACGTTTCCCGTTGAATATGGCTCATAACAGAACTTATTATTTCCTTCCTCTTTTCTACAGTATTTAAAGATACC
CCAAGAAGCTAATTATAACAAGACGAACTCCAATTCACTGTTCCTTGCATTCTAAAACCTTAAATACCAGAAAACAGCTTTTTCAAA
GTTGTTTTCAAAGTTGGCGTATAACATAGTATCGACGGAGCCGATTTTGAAACCGCGGTGATCACAGGCAGCAACGCTCTGTCATC
GTTACAATCAACATGCTACCCTCCGCGAGATCATCCGTGTTTCAAACCCGGCAGCTTAGTTGCCGTTCTTCCGAATAGCATCGGTAA
CATGAGCAAAGTCTGCCGCCTTACAACGGCTCTCCCGCTGACGCCGTCCCGGACTGATGGGCTGCCTGTATCGAGTGGTGATTTTG
TGCCGAGCTGCCGGTCGGGGAGCTGTTGGCTGGCTGGTGGCAGGATATATTGTGGTGTAAACATAACGAATTCGTCTCA

>R2

GGAGCAACCTTATTTTGTACTTTAAAAAATTCATTTTTTTTATTTTTTCGACTTTAAAGCCAAAATTATCCTTTATTTATGAAAGTGGA
TGTATTTTTATCCCTTTAATATAAGGTTGAGTTTTCTTTAAGTTTGTAATGTTAAGTGGGGCACTTTATATTAGTCCAATTAACAGAA
TTGACTTAGAAATTGATGGCGATTACTTGAAAAAATCGCTACAAAAACATAATAAAATAAAATTTCATTTGATTTTTAAACCAAAAG
AAAAGTGACATGACTAACGATTTGATCCCATCTTCTTTTTTTTTTAAGAAAAAAATTAAAAGAAAAATTTATTAAACTGGATGATTA
ATTTTAACATAAAAATTAAAAAAATAAAAATCAATCGAGCTCACTATTTGATATTATTATTTTCATCTTGATATTGTTACTTCATATCA
CTGAATTATAAAATGCGTTAACGGCAATTATCATAGACGCAGACTGAAAGCATAAAATTACAACTATTCCATTTTTGTTTTTTTTTTG
CCAACCCAAAAAAAACTAATAATTTATTTACATAATTATAGAAAATTGGAATTTATTCATGTTTTTGGACCATTCAGATAACCCCAC
AATAAAAAAAAAAGTGCACAAAAGTGCAAAGTGCTACGTGTCCTAATTATAGCCATACTACATTTGTGCATAAAATTAGCCACCATT
ATTTTTAAGATTATTCTTTTTCTTGTTTTAATTTATATGCCACACTTTCTTATTAATCCATCTTAAACAGAATGACACACTTTTTATTTG
ACAAATATTTAATGACATTATTAACATTTTATCCTCGTTAGATTTCACTATGCGGAATAGAGAATGTGTAAACTTTAAACTTCAAATT
TTGGCTCCGCATACGACTAGCGACTATATAATAATAGGAATTGAGCACTTGGCTTTTGTATATAGCTTCTATGTGTACCAAAATTAG
AAAATCAGGCGATTATTATAATCTTGTTGACTAAATATAGAATGCATCCATTACCCCCAAAAAGTGTGATTCCACTGTCATAGGAG
GTTTTTTTTATTTCATTTTATTTGTGCTTTCAATAATGTAGAGTAGTTTTACAAAGATCCTTTCTTTGTGACACATGGTAGGTAATATT
GCTGATTTTGTTGTAGTTTTGGGGTTATAAAGTTTCAAATTATTTATACTGGAGGGTAGGGGTGGGGGTTGTCTATAATGCAGGTT
ATGGTTTTACGTGAACTCAATAATTATTGTAGATACTAAGAAATCCACTCAGTGTTCTTGCGGTGTCTTGCTTTTGATTTCAGCATCA
CTTGTAGTTGATTGTGTTTAGATTATCACATTATTCTGTGGCTGTAACTGTATCCTTGTTAGTTGCTTTGTTTCTACACTGTTGTTTTC

CCTCTTTTATACCTATTTTGATATGTTGTACTCGAACGAGGGTCATCGGGGAACAACCTCTTTACCTCCGTGAGGTAGAGCTATGGT
CTGTGTCCACTCTACCCTCCCCAGATCCCTCTTGTAGGATTTCACTATATTGTAATATTAACTTGAGGTCACTATAGGAGCTCAAAAA
CTTCTAATTTTGAATCAATGTCTGGTTATACTTTTTTTGTCATAACTGTATCTCAAATGTGGTGTTTGGTTTATCTCATTTTGCAGAAG
TCAAGAAACAGGTTACTCCTGTTTGAGTGAGGAAAAGTTGGTTTGCCTGTCTGTGGTCTTTTTATAATCTTTTTCTACAGAAGAGAA
AGTGGGTAATTTTGTTTGAGAGTGGAAATATTCTCTAGTGGGAATCTACTAGGAGTAATTTATTTTCTATAAACTAAGTAAAGTTT
GGAAGGTGACAAAAAGAAAGACAAAAATCTTGGAATTGTTTTAGACAACCAAGGTTTTCTTGCTCAGAATGTCTGTTGCCTTGTTA
TGGGTTGTTTCTCCTTGTGACGTCTCAAATGGGACAAGTTTCATGGAATCAGTCCGGGAGGGAAACCGTTTTTTTGATTCATCGAG
GCATAGGAATTTGGTGTCCAATGAGAGAATCAATAGAGGTGGTGGAAAGCAAACTAATAATGGACGGAAATTTTCTGTACGGTCT
GCTATTTTGGCTACTCCATCTGGAGAACGGACGATGACATCGGAACAGATGGTCTATGATGTGGTTTTGAGGCAGGCAGCCTTGG
TGAAGAGGCAACTGAGATCTACCAATGAGTTAGAAGTGAAGCCGGATATACCTATTCCGGGGAATTTGGGCTTGTTGAGTGAAG
CATATGATAGGTGTGGTGAAGTATGTGCAGAGTATGCAAAGACGTTTAACTTAGGTTAGCTTCTTCAATCTATTCATTCGTTTACCA
AATATTATTTGGTAAGCACTAATTATGAATATATATATGTTCATGTTATTGATGAAGACAAAATTTGATCTTTGTTTGTTTATTCAGG
AACTATGCTAATGACTCCCGAGAGAAGAAGGGCTATCTAGGCAATATATGGTGAGGTTTCTAGCCATTTAATAACAGTTACGCGC
ACAAACACATATGATTAATCGGGGACGAGAAAAAAAGAAATGAAGTTTGAGTTTTGAGGGTCATATGTAATAGGTAAATCCGAG
CTTGACTAGCTTGAGATGTTTATTGTCATATCATGCTCAATACTAACCAAAACACTGAAAAAGAACTTGATTATATTTACATACTAA
TATTTTCATTTGCGTTGCTGTTCACATTTTTACCTATGGAACTGGTTTTTGTGATTTGTTATACTTCATATTCGATGTTAATAAAATAT
ATCATTCCTCCCTTTTTCTCCACTTCAAGCTTTACTGTAGTGTTGAAAGGGGAAACTCCTTTTAATGATTGCATATATAAACGAACTT
CTTGAGTTGAATAGTTTCTCATTATGATCTGTTTAAACAGTATGGTGCAGAAGAACAGATGAACTTGTTGATGGCCCAAACGCATC
ATATATTACCCCGGCAGCCTTAGATAGGTGGGAAAATAGGCTAGAAGATGTTTTCAATGGGCGGCCATTTGACATGCTCGATGGT
GCTTTGTCCGATACAGTTTCTAACTTTCCAGTTGATATTCAGGTTAGTCTACCAATTCTATGGTCTTTATATTTGTTCAATTTGCGTTT
GATGTCACTTTTGCTGAGGGCTTTTCTAATAGCTTACTTCAGCCTAGCGGAAATGTTTGTAGTTGAATCTCTAGTTCTGTCTCCTATA
TCTGTTTCTCTCGTCCTAGATACTACACATACTTCATTTCTGTTTTAACATTTTATTCGTCTTTTGGTGTTGTTTTGTATGTGAATCATA
TATTTGGAACAGAATCATTATTAGTTCACATGATTTCATTTGCTTTCTTCAATAGCGTAATTGTCTAACCTTCCAATATATGTTGCAG
CCATTCAGAGATATGATTGAAGGAATGCGTATGGACTTGAGAAAATCGAGATACAAAAACTTCGACGAACTATACCTTTATTGTTA
TTATGTTGCTGGTACGGTTGGGTTGATGAGTGTTCCAATTATGGCGCTGTCATGAGACGAATTCTGACAGGATATATTGGCGGGT
AAACCTAAGAGAAAAGAGCGTTTATTAGAATAATCGGATATTTAAAAGGGCGTGAAAAGGTTTATCCGTTCGTCCATTTGTATGTG
CATGCCAACCACAGGGTTCCCCTCGGGATCAAAGTACTTTGATCCAACCCCTCCGCTGCTATAGTGCAGTCGGCTTCTGACGTTCA
GTGCAGCCGTCATCTGAAAACGACATGTCGCACAAGTCCTAAGTTACGCGACAGGCTGCCGCCCTGCCCTTTTCCTGGCGTTTTCT
TGTCGCGTGTTTTAGTCGCATAAAGTAGAATACTTGCGACTAGAACCGGAGACATTACGCCATGAACAAGAGCGCCGCCGCTGGC
CTGCTGGGCTATGCCCGCGTCAGCACCGACGACCAGGACTTGACCAACCAACGGGCCGAACTGCACGCGGCCGGCTGCACCAAG
CTGTTTTCCGAGAAGATCACCGGCACCAGGCGCGACCGCCCGGAGCTGGCCAGGATGCTTGACCACCTACGCCCTGGCGACGTTG
TGACAGTGACCAGGCTAGACCGCCTGGCCCGCAGCACCCGCGACCTACTGGACATTGCCGAGCGCATCCAGGAGGCCGGCGCGG
GCCTGCGTAGCCTGGCAGAGCCGTGGGCCGACACCACCACGCCGGCCGGCCGCATGGTGTTGACCGTGTTCGCCGGCATTGCCG
AGTTCGAGCGTTCCCTAATCATCGACCGCACCCGGAGCGGGCGCGAGGCCGCCAAGGCCCGAGGCGTGAAGTTTGGCCCCCGCC
CTACCCTCACCCCGGCACAGATCGCGCACGCCCGCGAGCTGATCGACCAGGAAGGCCGCACCGTGAAAGAGGCGGCTGCACTGC
TTGGCGTGCATCGCTCGACCCTGTACCGCGCACTTGAGCGCAGCGAGGAAGTGACGCCCACCGAGGCCAGGCGGCGCGGTGCCT
TCCGTGAGGACGCATTGACCGAGGCCGACGCCCTGGCGGCCGCCGAGAATGAACGCCAAGAGGAACAAGCATGAAACCGCACC
AGGACGGCCAGGACGAACCGTTTTTCATTACCGAAGAGATCGAGGCGGAGATGATCGCGGCCGGGTACGTGTTCGAGCCGCCCG
CGCACCTCTCAACCGTGCGGCTGCATGAAATCCTGGCCGGTTTGTCTGATGCCAAGCTGGCGGCCTGGCCGGCCAGCTTGGCCGC
TGAAGAAACCGAGCGCCGCCGTCTAAAAAGGTGATGTGTATTTGAGTAAAACAGCTTGCGTCATGCGGTCGCTGCGTATATGATC
CGATGAGTAAATAAACAAATACGCAAGGGGAACGCATGAAGGTTATCGCTGTACTTAACCAGAAAGGCGGGTCAGGCAAGACGA
CCATCGGAACCCATCTAGCCCGCGCCCTGCAACTCGCCGGGGCCGATGTTCTGTTAGTCGATTCCGATCCCCAGGGCAGTGCCCGC
GATTGGGCGGCCGTGCGGGAAGATCAACCGCTAACCGTTGTCGGCATCGACCGCCCGACGATTGACCGCGACGTGAAGGCCATC
GGCCGGCGCGACTTCGTAGTGATCGACGGAGCGCCCCAGGCGGCGGACTTGGCTGTGTCCGCGATCAAGGCAGCCGACTTCGTG
CTGATTCCGGTGCAGCCAAGCCCTTACGACATATGGGCCACCGCCGACCTGGTGGAGCTGGTTAAGCAGCGCATTGAGGTCACGG
ATGGAAGGCTACAAGCGGCCTTTGTCGTGTCGCGGGCGATCAAAGGCACGCGCATCGGCGGTGAGGTTGCCGAGGCGCTGGCC
GGGTACGAGCTGCCCATTCTTGAGTCCCGTATCACGCAGCGCGTGAGCTACCCAGGCACTGCCGCCGCCGGCACAACCGTTCTTG
AATCAGAACCCGAGGGCGACGCTGCCCGCGAGGTCCAGGCGCTGGCCGCTGAAATTAAATCAAAACTCATTTGAGTTAATGAGG
TAAAGAGAAAATGAGCAAAAGCACAAACACGCTAAGTGCCGGCCGTCCGAGCGCACGCAGCAGCAAGGCTGCAACGTTGGCCA
GCCTGGCAGACACGCCAGCCATGAAGCGGGTCAACTTTCAGTTGCCGGCGGAGGATCACACCAAGCTGAAGATGTACGCGGTAC
GCCAAGGCAAGACCATTACCGAGCTGCTATCTGAATAGATCGCGCAGCTACCAGAGTAAATGAGCAAATGAATAAATGAGTAGAT

GAATTTTAGCGGCTAAAGGAGGCGGCATGGAAAATCAAGAACAACCAGGCACCGACGCCGTGGAATGCCCCATGTGTGGAGGA
ACGGGCGGTTGGCCAGGCGTAAGCGGCTGGGTTGTCTGCCGGCCCTGCAATGGCACTGGAACCCCCAAGCCCGAGGAATCGGCG
TGACGGTCGCAAACCATCCGGCCCGGTACAAATCGGCGCGGCGCTGGGTGATGACCTGGTGGAGAAGTTGAAGGCCGCGCAGG
CCGCCCAGCGGCAACGCATCGAGGCAGAAGCACGCCCCGGTGAATCGTGGCAAGCGGCCGCTGATCGAATCCGCAAAGAATCCC
GGCAACCGCCGGCAGCCGGTGCGCCGTCGATTAGGAAGCCGCCCAAGGGCGACGAGCAACCAGATTTTTTCGTTCCGATGCTCTA
TGACGTGGGCACCCGCGATAGTCGCAGCATCATGGACGTGGCCGTTTTCCGTCTGTCGAAGCGTGACCGACGAGCTGGCGAGGT
GATCCGCTACGAGCTTCCAGACGGGCACGTAGAGGTTTCCGCAGGGCCGGCCGGCATGGCCAGTGTGTGGGATTACGACCTGGT
ACTGATGGCGGTTTCCCATCTAACCGAATCCATGAACCGATACCGGGAAGGGAAGGGAGACAAGCCCGGCCGCGTGTTCCGTCC
ACACGTTGCGGACGTACTCAAGTTCTGCCGGCGAGCCGATGGCGGAAAGCAGAAAGACGACCTGGTAGAAACCTGCATTCGGTT
AAACACCACGCACGTTGCCATGCAGCGTACGAAGAAGGCCAAGAACGGCCGCCTGGTGACGGTATCCGAGGGTGAAGCCTTGAT
TAGCCGCTACAAGATCGTAAAGAGCGAAACCGGGCGGCCGGAGTACATCGAGATCGAGCTAGCTGATTGGATGTACCGCGAGAT
CACAGAAGGCAAGAACCCGGACGTGCTGACGGTTCACCCCGATTACTTTTTGATCGATCCCGGCATCGGCCGTTTTCTCTACCGCC
TGGCACGCCGCGCCGCAGGCAAGGCAGAAGCCAGATGGTTGTTCAAGACGATCTACGAACGCAGTGGCAGCGCCGGAGAGTTC
AAGAAGTTCTGTTTCACCGTGCGCAAGCTGATCGGGTCAAATGACCTGCCGGAGTACGATTTGAAGGAGGAGGCGGGGCAGGCT
GGCCCGATCCTAGTCATGCGCTACCGCAACCTGATCGAGGGCGAAGCATCCGCCGGTTCCTAATGTACGGAGCAGATGCTAGGGC
AAATTGCCCTAGCAGGGGAAAAAGGTCGAAAAGGACTCTTTCCTGTGGATAGCACGTACATTGGGAACCCAAAGCCGTACATTG
GGAACCGGAACCCGTACATTGGGAACCCAAAGCCGTACATTGGGAACCGGTCACACATGTAAGTGACTGATATAAAAGAGAAAA
AAGGCGATTTTTCCGCCTAAAACTCTTTAAAACTTATTAAAACTCTTAAAACCCGCCTGGCCTGTGCATAACTGTCTGGCCAGCGCA
CAGCCGAAGAGCTGCAAAAAGCGCCTACCCTTCGGTCGCTGCGCTCCCTACGCCCCGCCGCTTCGCGTCGGCCTATCGCGGCCGC
TGGCCGCTCAAAAATGGCTGGCCTACGGCCAGGCAATCTACCAGGGCGCGGACAAGCCGCGCCGTCGCCACTCGACCGCCGGCG
CCCACATCAAGGCACCCTGCCTCGCGCGTTTCGGTGATGACGGTGAAAACCTCTGACACATGCAGCTCCCGGTGACGGTCACAGC
TTGTCTGTAAGCGGATGCCGGGAGCAGACAAGCCCGTCAGGGCGCGTCAGCGGGTGTTGGCGGGTGTCGGGGCGCAGCCATGA
CCCAGTCACGTAGCGATAGCGGAGTGTATACTGGCTTAACTATGCGGCATCAGAGCAGATTGTACTGAGAGTGCACCATATGCGG
TGTGAAATACCGCACAGATGCGTAAGGAGAAAATACCGCATCAGGCGCTCTTCCGCTTCCTCGCTCACTGACTCGCTGCGCTCGGT
CGTTCGGCTGCGGCGAGCGGTATCAGCTCACTCAAAGGCGGTAATACGGTTATCCACAGAATCAGGGGATAACGCAGGAAAGAA
CATGTGAGCAAAAGGCCAGCAAAAGGCCAGGAACCGTAAAAAGGCCGCGTTGCTGGCGTTTTTCCATAGGCTCCGCCCCCCTGAC
GAGCATCACAAAAATCGACGCTCAAGTCAGAGGTGGCGAAACCCGACAGGACTATAAAGATACCAGGCGTTTCCCCCTGGAAGC
TCCCTCGTGCGCTCTCCTGTTCCGACCCTGCCGCTTACCGGATACCTGTCCGCCTTTCTCCCTTCGGGAAGCGTGGCGCTTTCTCATA
GCTCACGCTGTAGGTATCTCAGTTCGGTGTAGGTCGTTCGCTCCAAGCTGGGCTGTGTGCACGAACCCCCCGTTCAGCCCGACCGC
TGCGCCTTATCCGGTAACTATCGTCTTGAGTCCAACCCGGTAAGACACGACTTATCGCCACTGGCAGCAGCCACTGGTAACAGGAT
TAGCAGAGCGAGGTATGTAGGCGGTGCTACAGAGTTCTTGAAGTGGTGGCCTAACTACGGCTACACTAGAAGGACAGTATTTGG
TATCTGCGCTCTGCTGAAGCCAGTTACCTTCGGAAAAAGAGTTGGTAGCTCTTGATCCGGCAAACAAACCACCGCTGGTAGCGGT
GGTTTTTTTGTTTGCAAGCAGCAGATTACGCGCAGAAAAAAAGGATCTCAAGAAGATCCTTTGATCTTTTCTACGGGGTCTGACGC
TCAGTGGAACGAAAACTCACGTTAAGGGATTTTGGTCATGCATTCTAGGTGATTAGAAAAACTCATCGAGCATCAAATGAAACTG
CAATTTATTCATATCAGGATTATCAATACCATATTTTTGAAAAAGCCGTTTCTGTAATGAAGGAGAAAACTCACCGAGGCAGTTCCA
TAGGATGGCAAGATCCTGGTATCGGTCTGCGATTCCGACTCGTCCAACATCAATACAACCTATTAATTTCCCCTCGTCAAAAATAAG
GTTATCAAGTGAGAAATCACCATGAGTGACGACTGAATCCGGTGAGAATGGCAAAAGTTTATGCATTTCTTTCCAGACTTGTTCAA
CAGGCCAGCCATTACGCTCGTCATCAAAATCACTCGCATCAACCAAACCGTTATTCATTCGTGATTGCGCCTGAGCGAGTCGAAAT
ACGCGATCGCTGTTAAAAGGACAATTACAAACAGGAATCGAATGCAACCGGCGCAGGAACACTGCCAGCGCATCAACAATATTTT
CACCTGAATCAGGATATTCTTCTAATACCTGGAATGCTGTTTTCCCTGGGATCGCAGTGGTGAGTAACCATGCATCATCAGGAGTA
CGGATAAAATGCTTGATGGTCGGAAGAGGCATAAATTCCGTCAGCCAGTTTAGTCTGACCATCTCATCTGTAACATCATTGGCAAC
GCTACCTTTGCCATGTTTCAGAAACAACTCTGGCGCATCGGGCTTCCCATACAATCGGTAGATTGTCGCACCTGATTGCCCGACATT
ATCGCGAGCCCATTTATACCCATATAAATCAGCATCCATGTTGGAATTTAATCGCGGCCTTGAGCAAGACGTTTCCCGTTGAATATG
GCTCATAACAGAACTTATTATTTCCTTCCTCTTTTCTACAGTATTTAAAGATACCCCAAGAAGCTAATTATAACAAGACGAACTCCAA
TTCACTGTTCCTTGCATTCTAAAACCTTAAATACCAGAAAACAGCTTTTTCAAAGTTGTTTTCAAAGTTGGCGTATAACATAGTATCG
ACGGAGCCGATTTTGAAACCGCGGTGATCACAGGCAGCAACGCTCTGTCATCGTTACAATCAACATGCTACCCTCCGCGAGATCAT
CCGTGTTTCAAACCCGGCAGCTTAGTTGCCGTTCTTCCGAATAGCATCGGTAACATGAGCAAAGTCTGCCGCCTTACAACGGCTCT
CCCGCTGACGCCGTCCCGGACTGATGGGCTGCCTGTATCGAGTGGTGATTTTGTGCCGAGCTGCCGGTCGGGGAGCTGTTGGCTG
GCTGGTGGCAGGATATATTGTGGTGTAAACATAACGAATTCGTCTCA
